# Latent traits of lung tissue patterns in former smokers derived by dual channel deep learning in computed tomography images

**DOI:** 10.1038/s41598-021-84547-5

**Published:** 2021-03-01

**Authors:** Frank Li, Jiwoong Choi, Chunrui Zou, John D. Newell, Alejandro P. Comellas, Chang Hyun Lee, Hongseok Ko, R. Graham Barr, Eugene R. Bleecker, Christopher B. Cooper, Fereidoun Abtin, Igor Barjaktarevic, David Couper, MeiLan Han, Nadia N. Hansel, Richard E. Kanner, Robert Paine, Ella A. Kazerooni, Fernando J. Martinez, Wanda O’Neal, Stephen I. Rennard, Benjamin M. Smith, Prescott G. Woodruff, Eric A. Hoffman, Ching-Long Lin

**Affiliations:** 1 Department of Biomedical Engineering, University of Iowa, Iowa City, IA USA; 2 IIHR-Hydroscience and Engineering, 2406 Seamans Center for the Engineering Art and Science, University of Iowa, Iowa City, IA 52242 USA; 3 Department of Mechanical Engineering, University of Iowa, Iowa City, IA USA; 4 Department of Internal Medicine, School of Medicine, University of Kansas, Kansas City, KS USA; 5 Department of Radiology, University of Iowa, Iowa City, IA USA; 6 Department of Internal Medicine, University of Iowa, Iowa City, IA USA; 7 Department of Radiology, Seoul National University, Seoul, Republic of Korea; 8 Department of Radiology, Chungnam National University Sejong Hospital, Sejong, Republic of Korea; 9 Mailman School of Public Health, Columbia University, New York, NY USA; 10 Department of Medicine, University of Arizona, Tucson, AZ USA; 11 Department of Physiology, UCLA, Los Angeles, CA USA; 12 Department of Medicine, UCLA, Los Angeles, CA USA; 13 Department of Biostatistics, University of North Carolina, Chapel Hill, NC USA; 14 Department of Internal Medicine, University of Michigan, Ann Arbor, MI USA; 15 School of Medicine, Johns Hopkins, Baltimore, MD USA; 16 School of Medicine, University of Utah, Salt Lake City, UT USA; 17 Department of Radiology, University of Michigan, Ann Arbor, MI USA; 18 Weill Cornell Medicine, Cornell University, New York, NY USA; 19 School of Medicine, University of North Carolina, Chapel Hill, NC USA; 20 Department of Internal Medicine, University of Nebraska College of Medicine, Omaha, NE USA; 21 Department of Medicine, Columbia University, New York, NY USA; 22 Research Institute, McGill University Health Center, Montreal, Canada; 23 School of Medicine, UCSF, San Francisco, CA USA

**Keywords:** Chronic obstructive pulmonary disease, Biomedical engineering

## Abstract

Chronic obstructive pulmonary disease (COPD) is a heterogeneous disease and the traditional variables extracted from computed tomography (CT) images may not be sufficient to describe all the topological features of lung tissues in COPD patients. We employed an unsupervised three-dimensional (3D) convolutional autoencoder (CAE)-feature constructor (FC) deep learning network to learn from CT data and derive tissue pattern-clusters jointly. We then applied exploratory factor analysis (EFA) to discover the unobserved latent traits (factors) among pattern-clusters. CT images at total lung capacity (TLC) and residual volume (RV) of 541 former smokers and 59 healthy non-smokers from the cohort of the SubPopulations and Intermediate Outcome Measures in the COPD Study (SPIROMICS) were analyzed. TLC and RV images were registered to calculate the Jacobian (determinant) values for all the voxels in TLC images. 3D Regions of interest (ROIs) with two data channels of CT intensity and Jacobian value were randomly extracted from training images and were fed to the 3D CAE-FC model. 80 pattern-clusters and 7 factors were identified. Factor scores computed for individual subjects were able to predict spirometry-measured pulmonary functions. Two factors which correlated with various emphysema subtypes, parametric response mapping (PRM) metrics, airway variants, and airway tree to lung volume ratio were discriminants of patients across all severity stages. Our findings suggest the potential of developing factor-based surrogate markers for new COPD phenotypes.

## Introduction

Chronic obstructive pulmonary disease (COPD) is a complex and heterogeneous condition characterized by airflow limitation and was the fourth leading cause of death in the United States in 2016 and 2017^1,2^. Pulmonary function tests (PFTs) are commonly used to measure forced expiratory volume in one second (FEV_1_) and forced vital capacity (FVC) for the diagnosis of COPD, but spirometry is known to be poorly correlated with symptoms^3^. Furthermore, these measures cannot detect local structural and functional alterations in early stages of COPD. Thus, several studies have utilized imaging-based variables extracted from computed tomography (CT) lung images to identify COPD phenotypes. For example, since the introduction of the parametric response map (PRM)^4^ that matches CT images at inspiration and expiration to identify voxels of functional small airway disease (fSAD; i.e. non-emphysematous air-trapping region where CT intensity is less than − 856 HU on RV image) and emphysema (Emph; i.e. emphysematous region where CT intensity is less than − 950 HU on TLC image), a number of studies have focused on small airways using this voxel-wise imaging analysis technique^5^. To account for the topological features of PRM’s metrics, topological fSAD and Emph measures have been developed based on Minkowski functionals^6^. Haghighi et al. employed CT imaging-based structural and functional variables at both local and global scales to identify clinically relevant clusters for current and former smokers, respectively^7,8^.

Subjective selections of imaging-based variables may not be inclusive enough to describe all the features of COPD lungs^9^ because they are based on known physiology and pathophysiology. On the other hand, machine learning allows for identification of unknown disease phenotypes from CT images. In recent years, machine learning techniques, including traditional machine learning and contemporary deep learning techniques, have been used to discover new COPD phenotypes in order to provide reliable guidance in diagnoses and treatments^10–15^. However, these techniques are either labor intensive in image labeling, subjective in image labeling, or difficult in interpretation of how the machine learning models make decisions.

In medicine, syndromes are sets of symptoms that often occur together and can be modeled as latent traits (unobserved underlying disorders)^16^. It is hypothesized that there exist some latent traits behind the observed imaging features (detected by human and/or artificial intelligence), which are able to explain the heterogeneities of COPD. Therefore, in this study, we proposed an unsupervised three-dimensional (3D) convolutional autoencoder (CAE) model integrated with a feature constructor (FC) as a classifier (a CAE-FC model) to identify lung tissue pattern-clusters from one-dimensional (1D) representations (embeddings) of 3D regions of interests (ROIs) extracted from CT images of former smokers. We then applied an exploratory factor analysis (EFA) to explore the latent traits (factors) among these pattern-clusters and further assessed the capability of factors in predicting or diagnosing major clinical characteristics of COPD patients. Furthermore, these factors could be employed for unbiased sensitive subtyping of COPD progression without using human pre-defined features in the future.

## Results

A total of 541 former smokers and 59 healthy non-smokers (Stratum 1) from the SPIROMICS participants were analyzed. The demographic data and PFT measures for each stratum are shown in Table 1.

**Table 1:** Demographic data and PFT measures for COPD patients in each stratum. Entries are mean (SD) unless otherwise specified.

	Stratum 1	Stratum 2	Stratum 3	Stratum 4
	N = 59	N = 255	N = 156	N = 130
**Demographics**				
Age, years	53.83	66.07	68.76	65.75
	(9.54)	(7.37)	(6.24)	(6.98)
BMI, kg/m^2^	28.87	29.86	28.82	27.38
	(5.60)	(5.02)	(5.09)	(4.96)
Sex (male %)	27	42	60	47
Race, Caucasian/African American/Other (%)	62.71/30.51/6.78	83.97/9.61/6.42	87.45/9.02/3.53	86.92/11.54/1.54
GOLD 0/1/2/3/4 (%)	NA	96.15/1.92/1.92/0/0	0.39/38.43/61.18/0/0	0/0/0/70.77/29.23
**Maximal lung function**				
FEV_1_% predicted	100	95	76	35
	(13)	(14)	(16)	(9)
FEV_1_/FVC × 100%	81	77	58	37
	(5)	(5)	(8)	(9)

### Prediction of pulmonary function using factors

**Figure 1:**
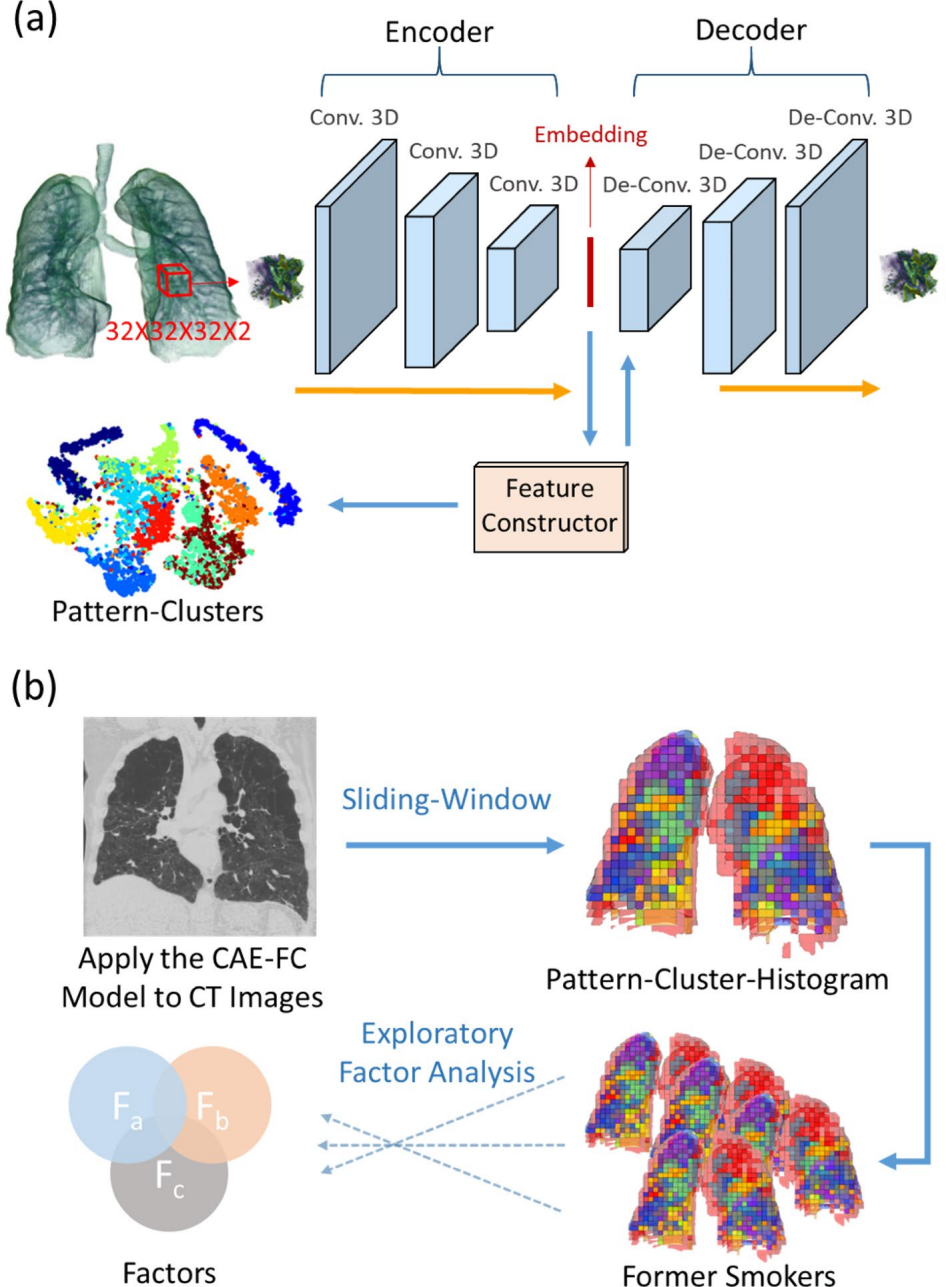
(**a**) 3D ROIs are randomly extracted from the 3D CT lung image and fed into the 3D convolutional autoencoder (CAE) to learn the 1D representations (embedding) of the ROIs. A feature constructor (FC) uses the embedding to generate pattern-clusters. (**b**) The CAE-FC model is applied to the ROIs extracted by the sliding-window technique to classify lung tissue patterns and construct a pattern-cluster-histogram for each subject. Exploratory factor analysis (EFA) is then used to extract the latent traits from the pattern-cluster-histograms of the subjects.

We first trained the CAE-FC model by increasing the number of embeddings (which is the same as the number of pattern-clusters) from 16 to 112 with an increment of 16. Then the optimal CAE-FC model was used to generate a pattern-cluster histogram for each subject and EFA was employed to extract factors from all of the histograms (Fig. 1). Finally, a multivariable regression model was developed to explore the relationship between the derived factor scores and associated PFT measures for all the subjects. The optimal number of pattern-clusters was chosen based on its ability to predict pulmonary function. We calculated the Akaike Information Criterion (AIC) values for different models set up by different numbers of embeddings.

The optimal model consisted of 80 embeddings and seven factors (F0–F6), which yielded the lowest AICs for both FEV_1_% predicted (− 476.02) and FEV_1_/FVC (− 590.98) (Table E3). The results demonstrated the capability of factors in predicting pulmonary function. On the test dataset with 135 subjects, the coefficient of determination (R^2^) between the true values and predicted values at the baseline visit reached 0.66 and 0.71 for FEV_1_% predicted and FEV_1_/FVC, respectively (Figure E4). To evaluate the effect of adding Jacobian as the second data channel, another CAE-FC model with 80 pattern-clusters was trained using TLC ROIs with CT intensity only. The 3-factors predictive models with the 80 pattern-clusters yielded AICs of − 417.69 and − 531.25 for FEV_1_% predicted (R^2^ = 0.42) and FEV_1_/FVC (R^2^ = 0.50), respectively (Table E3 and Figure E4).

Welch’s ANOVA was used in comparing the mean factor scores among the seven factors. The results demonstrated significant differences between healthy non-smokers and subjects with different severities classified by the criteria of Global Initiative for Chronic Obstructive Lung Disease (GOLD 0–4)^17^ for F0, F2, F4, F5, and F6 (Table E4). However, the post-hoc pairwise comparisons were conducted only on F0 and F4 since their effect sizes were deemed to be a close-to-medium effect or a large effect by Cohen’s d criteria^18,19^ (Table E12). For F0, the only pairwise comparison that did not demonstrate a significant difference was from the pair of GOLD 3 subjects and GOLD 4 subjects (Table E5). As for F4, the comparison pairs that did not demonstrate significant differences were from the pair of GOLD 0 subjects and healthy non-smokers, the pair of GOLD 0 subjects and GOLD 1 subjects, the pair of GOLD 0 subjects and GOLD 2 subjects, the pair of GOLD 1 subjects and healthy non-smokers, the pair of GOLD 1 subjects and GOLD 2 subjects, and the pair of GOLD 2 and healthy non-smokers (Table E6).

### Comparison of predictabilities of factors, pattern-clusters, and imaging-based variables

Several multivariable regression models were built to examine the relationships between the predictors (factors, pattern-clusters, or imaging-based metrics) and the criterion variables (FEV_1_% predicted and FEV_1_/FVC). AIC was used to assess the relative efficacy of the models. The results demonstrated that the multiple regression models based on factors and imaging metrics had lower AICs than those of pattern-clusters. Furthermore, the factor-based model (AIC = − 469.65) was a better predictor of FEV_1_% predicted than the imaging-metric-based model (AIC = − 462.73). On the other hand, the imaging-metric-based model (AIC = − 605.79) outperformed the factor-model (AIC = − 588.12) in predicting FEV_1_/FVC.

Regression models based on F0, F4, Emph%^8^ (the percentage of Emph voxels on TLC image), and fSAD%^8^ (the percentage of fSAD voxels on TLC image) were then built with forward feature selection applied. F0, F4, Emph%, and fSAD% were all preserved in the forward feature selection process. The combination models based on F0, F4, Emph%, and fSAD% were the best models to predict FEV_1_% predicted (AIC = − 481.14) and FEV_1_/FVC (AIC = − 624.50). The R^2^ values, the AIC values, and the numbers of predictors chosen by the forward feature selection method for various models are shown in Figure E5. The predictors chosen by the forward feature selection method for various models are listed in Table E7.

### Comparison of averaged CT intensities and Jacobians for factors

**Figure 2:**
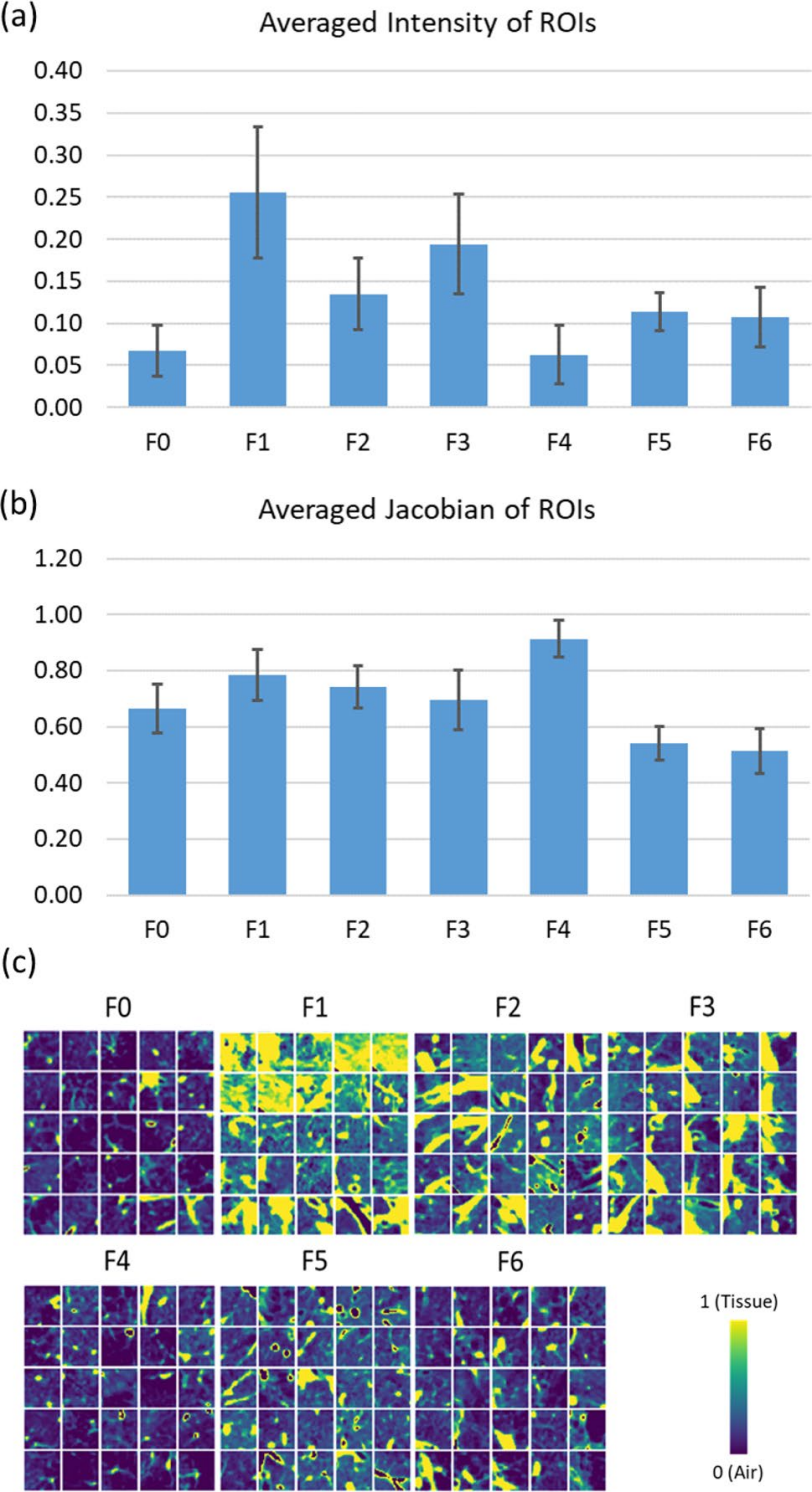
Bar charts showing (**a**) the averaged intensity of ROIs with large contribution to each factor and (**b**) the averaged Jacobian of ROIs with large contribution to each factor. Significant differences (p < 0.001) between factors were found in both the averaged intensity and the averaged Jacobian by Welch’s ANOVA. The pairwise Games–Howell post-hoc tests showed that all the pairs were significantly different except for the pair of F5 and F6 that was not significantly different for either the averaged intensity (p = 0.718) or the averaged Jacobian (p = 0.118). (**c**) Samples of the ROIs taken from the pattern-clusters with strong contribution to each factor.

**Figure 3:**
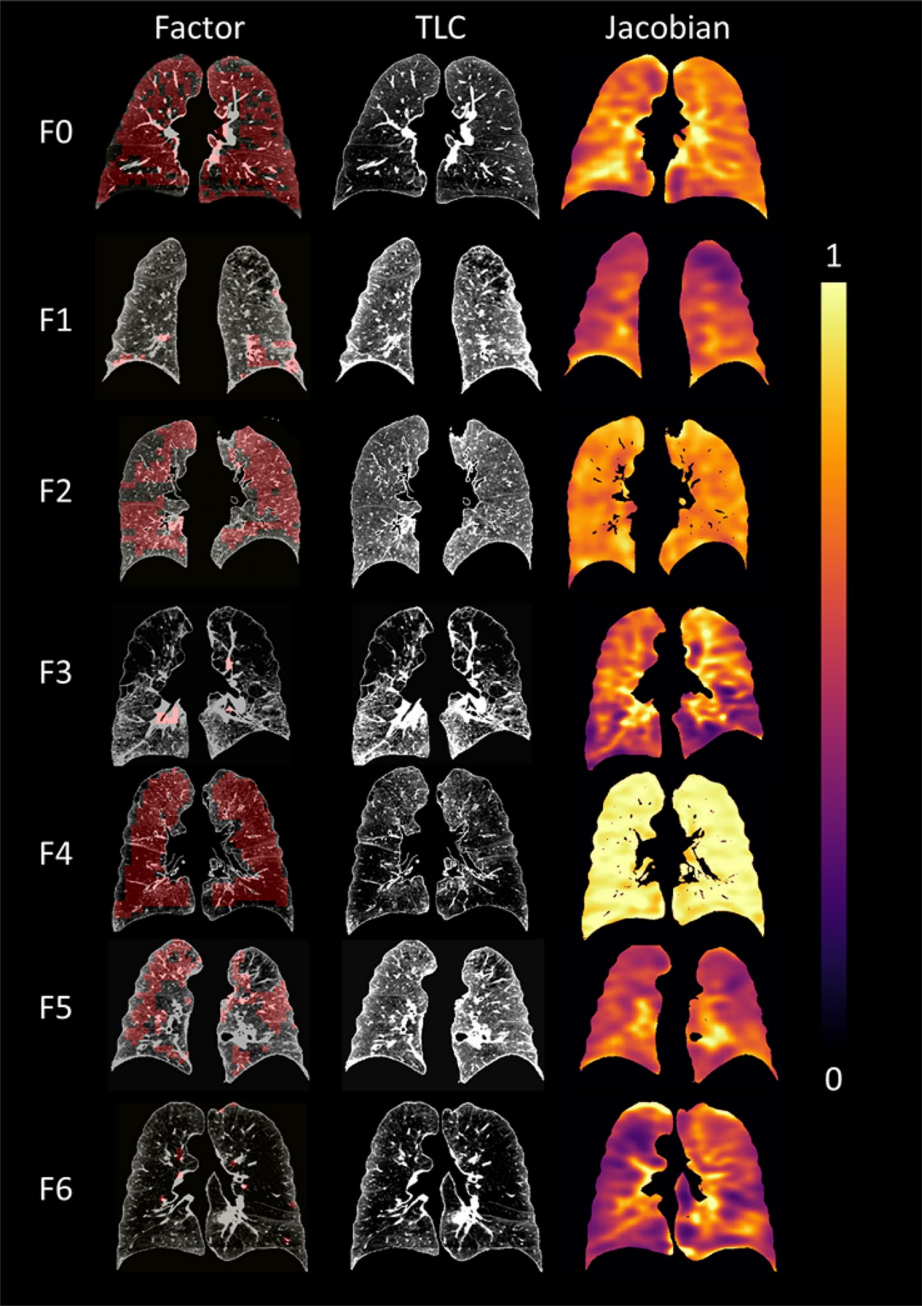
Factor-representative subjects (i.e. 99th percentile of the factor score among the subjects), shown in a coronal view (posterior–anterior). The pattern-clusters with strong contribution to each factor, ranging from F0 to F6, are shaded with red on the images in the left column. TLC images with CT intensities are displayed in the middle. Jacobian images are shown in the right column.

Significant differences (p < 0.001) between factors were found in both the averaged CT intensity and the averaged Jacobian of the ROIs belonging to each factor by Welch’s ANOVA (Fig. 2). The pairwise Games–Howell post-hoc tests demonstrated that all the pairs were significantly different except for the pair of F5 and F6 (Table E8). Generally, F0 and F4 had lower intensities whereas F1 and F3 had relatively higher intensities. As for the Jacobian, F4 had the least deformation whereas F5 and F6 had the largest deformation. Figure 2c displays the samples of pattern-clusters having large contribution to the factors. Figure 3 shows representative subjects for each factor along with the color-coded Jacobian values of the lungs.

### Emphysema subtypes and airway variants

**Figure 4:**
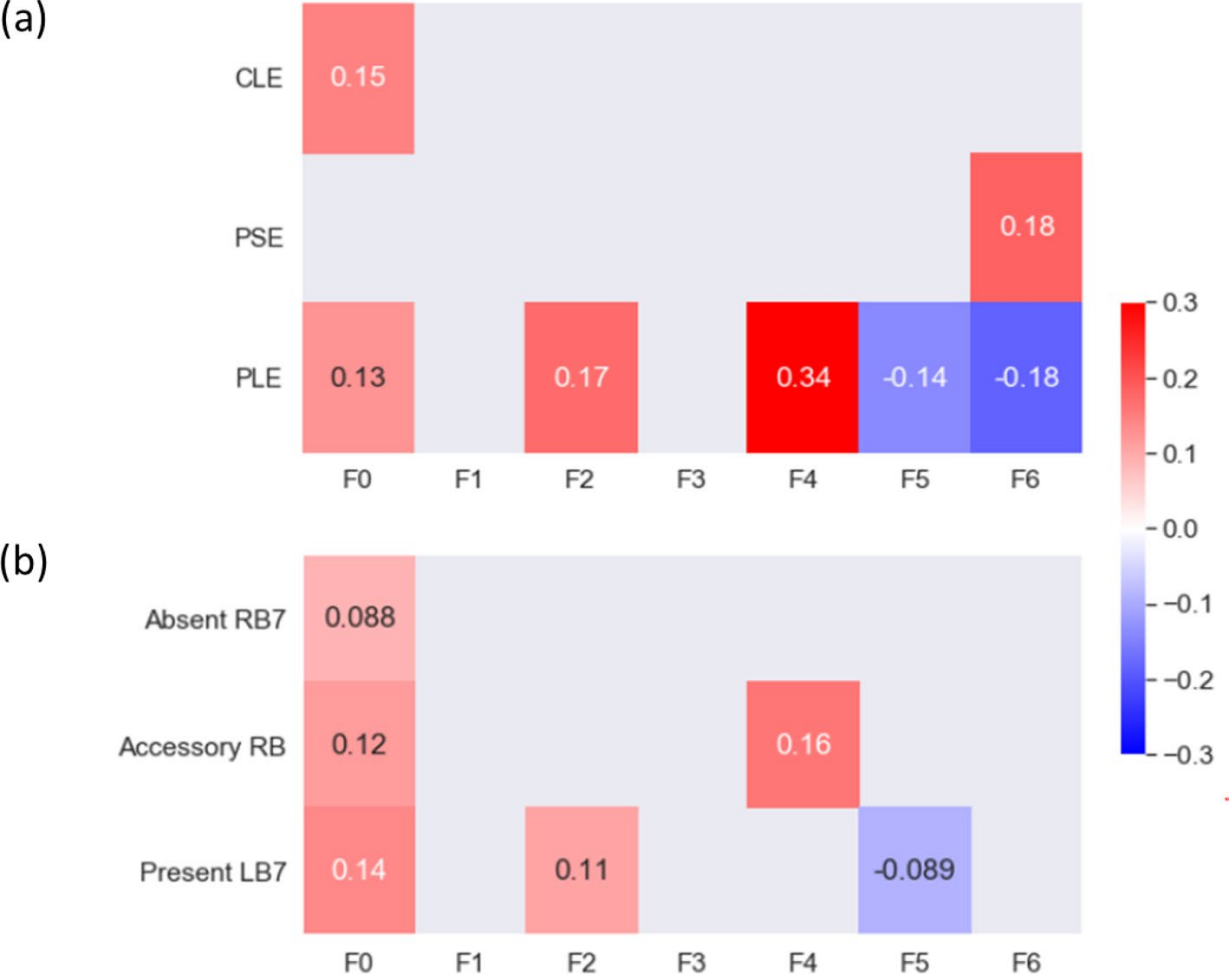
(**a**) The correlations between the factors and the emphysema subtypes. The subtypes are CLE, PLE, and PSE. (**b**) The correlations between the factors and the airway variants. The three variants are: (1) absence of a right medial-basal RB7 branch in RLL (Absent RB7), (2) presence of an accessory sub-superior RB branch in the RLL (Accessory RB), and (3) presence of an accessory LB7 branch in the LLL (Present LB7; see Figure E3). Only correlations with p value less than 0.05 are shown in the figure.

Experienced thoracic radiologists have carefully examined 1000 subjects in the SPIROMICS cohort and labeled them with three emphysema subtypes: centrilobular emphysema (CLE), panlobular emphysema (PLE) and paraseptal emphysema (PSE). Within those subjects, 253 of them overlapped with the former smokers used in this study. Bi-serial correlation was used to test the correlations between the factors and the emphysema subtypes (Fig. 4a). F4 was moderately correlated with PLE (r = 0.34). F0 and F2 were weakly correlated with PLE (r = 0.13 and 0.17, respectively), whereas F5 and F6 were negatively and weakly correlated with PLE. F0 was the only factor which had an association with CLE while F6 was the only factor which had an association with PSE.

The associations between the factors and the airway variants (central airway branch variation, a genetic risk factor for COPD; Figure E3) are shown in Fig. 4b. The percentages of standard anatomy and airway variants can be found in Table E9. F0 was weakly correlated with an accessory sub-superior branch in the right lower lobe (RLL; r = 0.12), a left medial basal LB7 branch in the left lower lobe (LLL; r = 0.14), and an absent right medial-basal RB7 branch (r = 0.088). F4 was also correlated with an accessory branch in the RLL (r = 0.16).

### Correlations of factors with clinical data, imaging-based variables, and medication use

**Figure 5:**
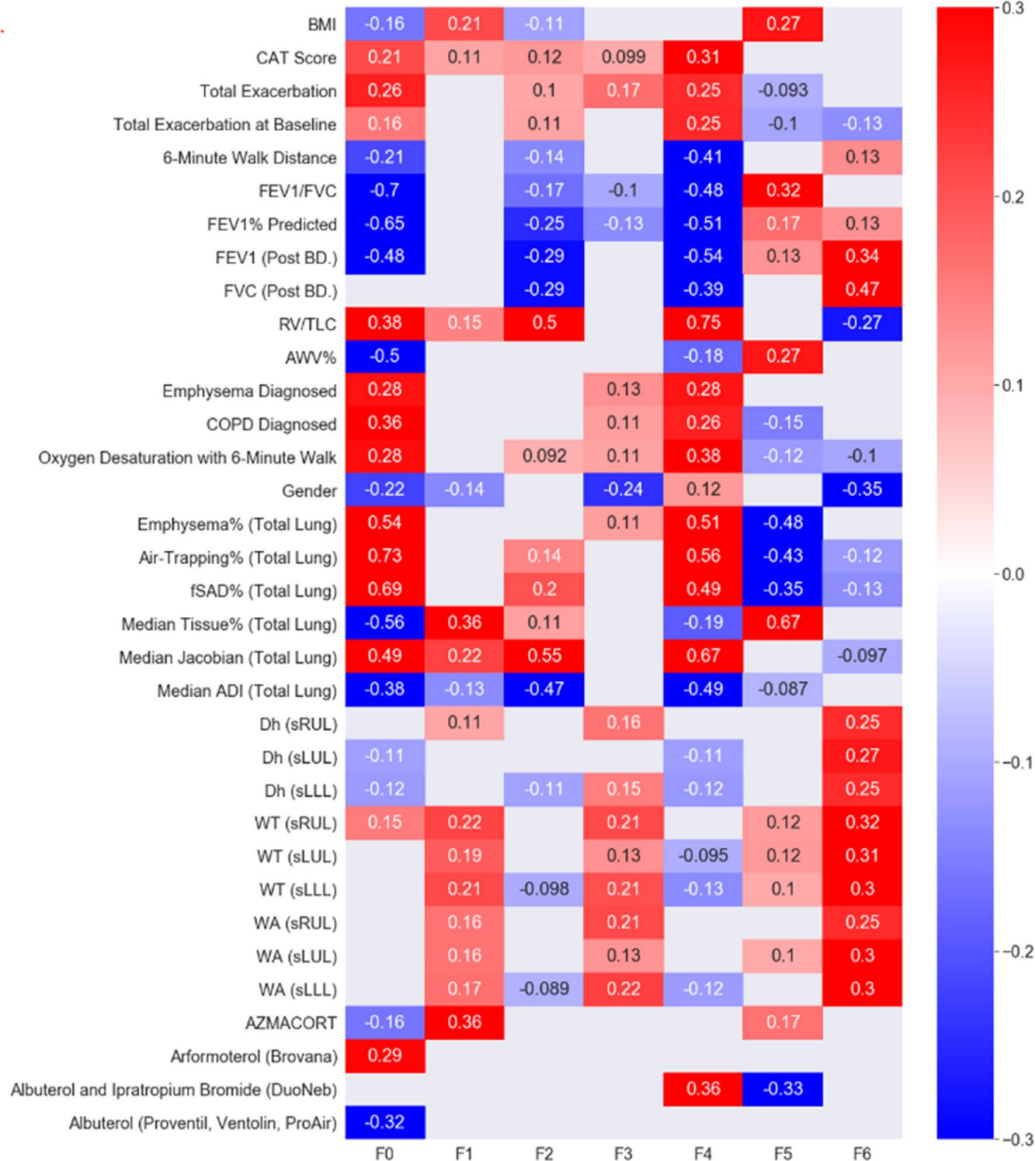
Correlations between each of the seven factors and related clinical data, imaging-based variables, and use of drugs. Only correlations with p value less than 0.05 are shown in the figure.

The correlation heat-map in Fig. 5 summarizes the correlations of seven factors with clinical data, imaging-based variables, and medication use. Only variables with moderate–high significant correlations with the factors are displayed in the heat-map. Significant and relevant variables for each factor are described below and more results can be found in Table E10.

F0 was negatively correlated with FEV_1_ (r = − 0.48), FEV_1_/FVC (r = − 0.7), and FEV_1_% predicted (r = − 0.65). However, it was not significantly correlated with FVC (r = − 0.048). Clinically, it showed moderate correlations with the diagnosis of COPD (r = 0.36), the diagnosis of emphysema (r = 0.28), and the total number of exacerbations (r = 0.26). As for imaging-based variables, it displayed strong correlations with Emph% (r = 0.54), fSAD% (r = 0.69) and AirT% (the percentage of air-trapped voxels in a RV CT scan; r = 0.73), Tissue %, (the median tissue fraction of the total lung; r = − 0.56), and AWV% (the airway tree to lung volume ratio; r = − 0.50) of the total lung. F0 also had moderate correlations with RV/TLC (CT-measured RV to TLC volume ratio; r = 0.38), the median Jacobian of the total lung (r = 0.49), and the median of a strain-based metric of the median anisotropic deformation index (ADI) of the total lung (r = − 0.38). Lastly, it was correlated negatively with the use of albuterol (r = − 0.32) and positively with the use of arformoterol (r = 0.29).

F1, F2, and F3 had few significant correlations with either of the PFT measures, clinical data, or medication use. F1 was correlated only with the median tissue% (r = 0.36). F2 had a moderately negative correlation with FEV_1_ (r = − 0.29), FEV_1_% predicted (r = − 0.25), and FVC (r = − 0.29). It was also correlated with the median Jacobian of the total lung (r = − 0.55), the median ADI of the total lung (r = − 0.47), and RV/TLC (r = 0.50). Female patients tended to have lower F3 (r = − 0.24).

F4 was negatively correlated with FEV_1_ (r = − 0.54), FVC (r = − 0.39), FEV_1_/FVC (r = − 0.48), and FEV_1_% predicted (r = − 0.54). Clinically, it had moderate correlations with the diagnosis of COPD (r = 0.26), the diagnosis of emphysema (r = 0.28), the total number of exacerbations (r = 0.25), the CAT score (r = 0.31), and the 6-min walk distance (r = − 0.41). As for imaging-based metrics, it was positively correlated with Emph% (r = 0.51), AirT% (r = 0.56), fSAD% (r = 0.49), and the median Jacobian of the total lung (r = 0.67), and negatively correlated with the median ADI of the total lung (r = − 0.49), and RV/TLC (r = − 0.75). In addition, it was correlated positively with the use of albuterol and ipratropium bromide (r = 0.36).

F5 had a moderate correlation with FEV_1_/FVC (r = 0.32) and BMI (r = 0.27). It was negatively correlated with Emph% (r = − 0.48), AirT% (r = − 0.43) and fSAD% (r = − 0.35), and positively correlated with median Tissue% (r = 0.67) and AWV% (r = 0.27). Furthermore, it was negatively correlated with the use of albuterol and ipratropium bromide (r = − 0.33).

F6 exhibited a moderate correlation with FEV_1_ (r = 0.34), FVC (r = 0.47), and RV/TLC (r = − 0.27). Female patients tended to have lower F6 (r = − 0.35). Moreover, F6 was associated with increased airway diameter, wall thickness, and airway wall area located in the LLL, left upper lobe (LUL), and right upper lobe (RUL).

### Factor-coded ROIs and Factor-based Histograms

**Figure 6:**
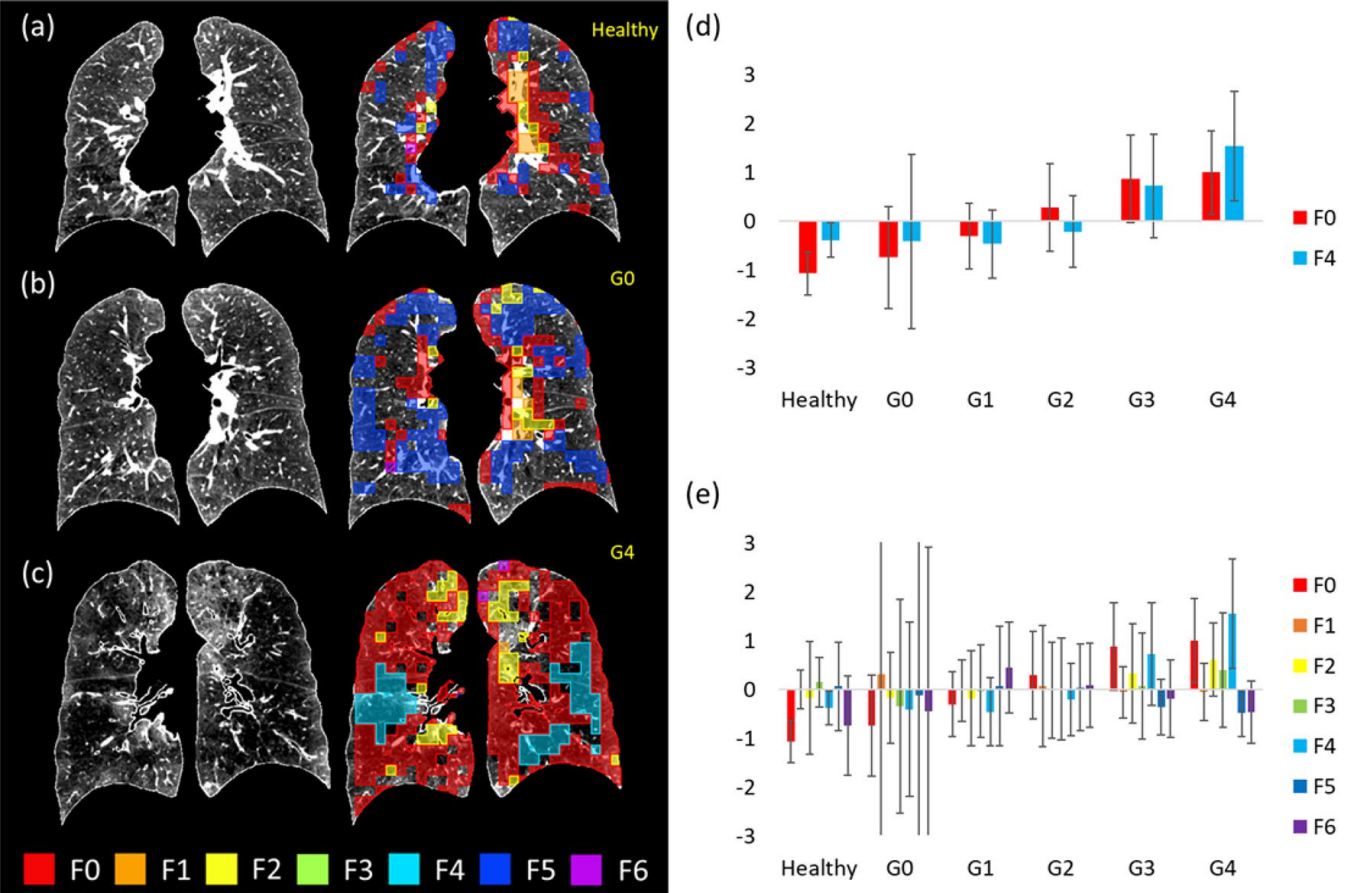
(**a**) The original TLC image (left) and the color-coded image (right) of a healthy non-smoker (FEV_1_/FVC = 79.02%, FEV_1_% predicted = 81.47%) whose factor scores of F0 and F4 are the closest to the means of the healthy non-smoker cohort. (**b**) The original TLC image (left) and the color-coded image (right) of a GOLD 0 subject (FEV_1_/FVC = 56%, FEV_1_% predicted = 97.88%) whose factor scores of F0 and F4 are the closest to the means of the GOLD 0 subjects. (**c**) The original TLC image (left) and the color-coded image (right) of a GOLD 4 subject (FEV_1_/FVC = 29.21%, FEV_1_% predicted = 20.05%) whose factor scores of F0 and F4 are the closest to the means of the GOLD 4 subjects. The images are plotted in a posterior–anterior view. (**d**) The averaged factor scores of F0 and F4 for the healthy non-smokers, and GOLD 0–GOLD 4 subjects. (**e**) The variations of the factor scores for the GOLD 0 subjects are the largest among all subgroups, implying the heterogeneous nature of the subjects at risk.

Figure 6a–c compare the factor maps of a healthy non-smoker, a GOLD 0 subject, and a GOLD 4 subject to illustrate the capability of factors in characterizing local tissue patterns. Figure 6d shows the histogram of F0 and F4 for healthy non-smokers and COPD subjects. F0 was able to differentiate healthy non-smokers and GOLD 0 patients as well as any pair of subjects at different levels of COPD severity except for GOLD 3 subjects and GOLD 4 subjects, whereas F4 could differentiate GOLD 3 subjects and GOLD 4 subjects. Figure 6e displays the histogram of all seven factors, showing that GOLD 0 subjects had the largest variation among all subjects.

## Discussion

Unsupervised machine learning of CT images has been applied to improve accuracy, efficiency, and reliability of emphysema subtype detection, thus alleviating the workload of radiologists. These studies relied on human-defined imaging filters or textons to extract features of images^10–12^. On the other hand, deep learning has the capability to detect features from images without human intervention. Using four slices of CT images in different directions as inputs, Gonzalez et al.^15^ trained a supervised convolutional neural network (CNN) deep learning model to predict COPD disease stage, lung function, mortality and exacerbation. Humphries et al.^13^ proposed a deep learning model with two-dimensional CNN and long short-term memory architectures (LSTM) combined together to classify Fleischner grade of emphysema. Deep learning is an effective approach to train a model for prediction, but the interpretability of the detected features is relatively low. The mechanism of how deep learning models form decisions is not straightforward. This is due in large part to the problem of subjective or incomprehensive imaging-based metrics and the difficulty to interpret deep learning-detected features. Singla et al.^14^ proposed a 3D CAE with attention mechanism that aggregated local image features to a subject-level representation for predicting COPD severity. The attention mechanism quantified the relevance of a region to the disease and thus enhanced the interpretability of the results. However, the attention mechanism was only allowed to provide interpretations associated with the severity and did not consider other features that may also contribute to the disease’s variability.

To address the issues above, this study aimed to develop an unsupervised deep-learning CNN model, which could identify latent traits (factors) from CT lung images of former smokers. Then, identified factors were correlated with existing known criterion variables. We identified seven salient factors that contributed substantially to the prediction of pulmonary functions and correlated significantly with the imaging-based and clinical variables, showing the feasibility and usefulness of the proposed CAE-FC and EFA framework. The factors are derived purely from CT images without human bias and could serve as features in subtyping of COPD progression in the future.

### Prediction of pulmonary function using factors

While models based on pattern-clusters, factors and imaging-based metrics were all comparable in predicting pulmonary function measured by spirometry, the number of predictors based on pattern-clusters was excessive compared to those of factors and imaging-based metrics. Furthermore, the combination models based on F0, F4, Emph%, and fSAD% outperformed the imaging-metric-based models. Given that F0, F4, Emph%, and fSAD% were all chosen by the forward feature selection method, the factors could explain additional variations of PFT measures besides those already explained by Emph% and fSAD%.

Previous studies have used four slices in three different directions per CT image as an input to train a convolutional deep learning model on 9983 smokers, which was able to achieve a strong coefficient of determination (R^2^ = 0.54) between true FEV_1_ and predicted FEV_1_^15^. The 3D CAE-FC model showed its prediction efficacy for lung function (R^2^ = 0.57 for FEV_1_; R^2^ = 0.66 for FEV_1_% predicted). Moreover, we demonstrated that the addition of Jacobian to the second channel of ROIs improved the explained variance by 23.9% and 20.7% for FEV_1_% predicted and FEV_1_/FVC, respectively. With Jacobian, we were able to account for lung deformation without explicitly learning using expiration images. While a network that learns directly from inspiration and expiration images could be constructed, the voxel-to-voxel correspondence between them (local lung deformation information) would be missing. As a result, the two important COPD phenotypes, PRM’s fSAD and Emph, would be not accounted for in learning, rendering the network ineffective. Additionally, PRM’s fSAD and Emph are two mutually exclusive voxel-wise scalar fields. The current CNN model does not rule out the possibility of new phenotypes such as those potentially associated with mixed fSAD/Emph 3D topological patterns to assess COPD.

Since the model was trained on CT images of GOLD 0–4 subjects, the fact that F0 identified the healthy control group as a distinct cohort from COPD patients not only validated our method, but also suggested the potential of F0 as a surrogate marker in detecting local abnormalities in susceptible subjects progressing from heathy to at risk. On the other hand, F4 was able to differentiate GOLD 3 subjects from GOLD 4 subjects, suggesting the potential of F4 as a surrogate marker in detecting the worsening of lung function (the rate of functional decline) in severe COPD subjects. While a total of seven factors were identified, two of them, F0 and F4, were sufficient to distinguish between the spirometric stages of COPD.

### Correlations of factors with clinical data, imaging-based variables and medication use

Female patients were found to have negative correlations with the factor scores in F3 and F6. Studies have shown that females may be more susceptible to developing COPD, probably because the airways of females are relatively smaller than those of males at the same lung volume, leading to greater concentration of tobacco smoke per unit area of small airway surface^20^. Our results also demonstrated that female patients tended to have smaller factor scores in F6 and they had smaller airways. Furthermore, F3 had a similar trend but not as strong as F6. This suggested that sex might contribute to COPD tissue phenotypes.

Moreover, we speculated that F6 was related to bronchodilator responsiveness (BDR). It has been reported that COPD patients with BDR had thicker airways on CT images^21^ and that FVC is more sensitive in BDR than FEV_1_ for patients with severe airflow obstruction^22^. These observations resemble our results that F6 was positively related to larger airway dimensions (including airway diameter, airway wall thickness, and airway wall area) and more correlated with FVC than FEV_1_. F6 also had some positive correlation with the use of short-acting bronchodilator (nebulized albuterol) (Table E10), which can relieve air-trapping. Since BDR is associated with a faster rate of lung function decline^23^, F6 may potentially serve as a surrogate marker of BDR to signify a risk of lung function decline.

Our findings also indicated that the use of inhaled bronchodilator medications might be related to the identified factors. F0 was correlated negatively with the use of a short-acting β-agonist (nebulized albuterol) in last 3 months, and positively with the use of a longer-acting β-agonist (arformoterol) in last 3 months. Short-acting β-agonists are used by patients with relatively few symptoms and low risk of COPD exacerbations in order to relieve acute breathlessness^24^. Long-acting bronchodilators are generally recommended for more severe patients^24^. This would explain why F0 was positively related to the use of a long-acting bronchodilator. On the other hand, F4 was positively correlated with albuterol combined with ipratropium bromide, suggesting that patients with higher F4 might need combination bronchodilators to control their symptoms^25^. The findings coincided with the higher correlations of F4 with the CAT score and the total number of exacerbations. The refined 2020 GOLD ABCD assessment tool comprises three steps: diagnosis (post-bronchodilator FEV_1_/FVC < 0.7), assessment of airflow limitation (GOLD 1–4) and assessment of symptoms/risk of exacerbations (group A–D). F4 seems to associate with GOLD 4, group D and recommended initial pharmacological treatment.

### Interpretations of factors

Lower intensities of the ROIs were found in both F0 and F4, suggesting more emphysema. F4 had greater Jacobian, suggesting less contractive lungs. While both F0 and F4 were highly correlated with emphysema (↑) and air-trapping (↑), the characteristics of F0 were AWV% (↓), tissue% (↓), diagnosis of COPD (↑), CLE (↑), and fSAD% (↑), and those of F4 were FVC (↓), six minute walk distance (↓), CAT score (↑), PLE (↑), Jacobian (↑) and RV/TLC (↑) (Table 2), where ↑(or↓) denotes an increase (or decrease). Due to the distinct characteristics between F0 and F4, it is speculated that they are two COPD subtypes which contribute significantly to the impairment of pulmonary function.

**Table 2:** Comparisons of the associations with known variables between F0 and F4. The numbers in the table represent the strength of correlation with the factors. An increment of the number represent a 0.1 difference of r.

	F0	F4
Tissue %	↓*6	
AWV %	↓*5	
Long-acting Bronchodilator	↑*3	
FEV_1_/FVC	↓*7	↓*4
fSAD %	↑*7	↑*4
FEV_1_% predicted	↓*7	↓*5
COPD	↑*4	↑*3
Emphysema %	↑*5	↑*5
FEV_1_	↓*5	↓*5
CAT Score	↑*2	↑*3
6 Min. Walk Distance	↓*2	↓*4
Jacobian	↑*5	↑*7
RV/TLC	↑*4	↑*8
FVC		↓*4
Combination of more than one bronchodilator		↑*4

F0 might be related to air-trapping due to small airway diseases because of its correlations with fSAD% (↑) and AWV% (↓). From the perspective of pulmonary function, F0 represents the classic feature of COPD: FEV_1_/FVC (↓) and FEV_1_% predicted (↓). It has been reported that functional small airway diseases and airway tree caliber to lung size ratio (a host factor) are associated with rate of FEV_1_ decline and COPD risk^5,26^. Additionally, F0 is able to differentiate between healthy non-smokers and former smokers at risk of COPD. Thus, F0 is potentially a latent trait that is associated with COPD risk and lung function decline.

As for F4, it is likely to capture air-trapping of a less contractive lung (Jacobian ↑) associated with decreased diaphragm mobility because of its correlations with FVC (↓) and RV/TLC (↑). Diaphragm mobility has been shown to have impact on dyspnea and exercise tolerance^27^, which explains why patients with greater F4 have more symptoms (CAT score ↑) and shorter six minute walk distance (↓). Recent studies have suggested that FVC is an important clinical marker which can differentiate clinical outcomes in COPD populations^28,29^, and RV/TLC is a predictor of rate of lung function decline^30^. Therefore, F4 is potentially a latent trait that is associated with symptoms and worsening of lung function.

According to the radiologists’ reading of emphysema subtypes, F0 was correlated with CLE, a subtype which could lead to respiratory bronchitis^31^, while F4 was correlated with PLE, a subtype which might cause a more inflated lung^32^. Therefore, F0 and F4 may represent two phenotypes which are dominated by airway disease and parenchymal disease, respectively^32^. In 2015, the Fleischner classification has replaced the terms “panlobular emphysema” with “confluent emphysema” and “advanced destructive emphysema (ADE)” for patients without alpha-1 antitrypsin deficiency (A1AD)^33^. Since patients with A1AD are rare (1.53%) in the current dataset, two experienced radiologists carefully reviewed the CT images of F0 and F4 from representative subjects (Fig. 3) and characterized their emphysema patterns following the statement of the Fleischner Society^33,34^. The major pattern-cluster masks of F0 were located relatively near central airways and might include vessels and small airways (Figure E6). Overall, no advanced lung parenchymal destruction is observed in F0 masks. On the other hand, the major pattern-cluster masks of F4 were found in peripheral lung regions and were more likely to include vessels rather than small airways (Figure E6). Advanced parenchymal destructions were observed in these regions, resembling the advanced emphysema patterns shown in the study by Lynch et al.^34^. That is, the patterns of F0 appear to be associated with airway diseases and early stages of emphysema. This is consistent with the observed association of F0 with obstruction characteristics such as decreased FEV_1_%, FEV_1_/FVC, and AWV%. In contrast, F4 appears to be associated with panlobular CT patterns for more advanced emphysema, being consistent with the observed negative correlation between F4 and FVC. A previous study suggested that CLE is associated with worse small airways remodeling and narrowing, having a different inflammatory process from PLE^35^. Thus, the radiologists’ reading confirmed that F0 and F4 could capture the major characteristics of different phenotypes.

Smith et al. reported that an accessory airway branch is associated with higher risks of COPD and bronchitis and an absent right medial-basal airway is associated with higher risks of COPD and dyspnea^36^. Our result demonstrated that airway variants were correlated with F0 and F4. This is particularly true for F0, which had a weak but significant association with airway variants of Accessory RB, Present LB7, and Absent RB7. Overall, our result suggested that the factors are able to detect the COPD susceptibility associated with genetically-determined airway branch variation (a genetic risk factor).

### A post hoc study

**Figure 7:**
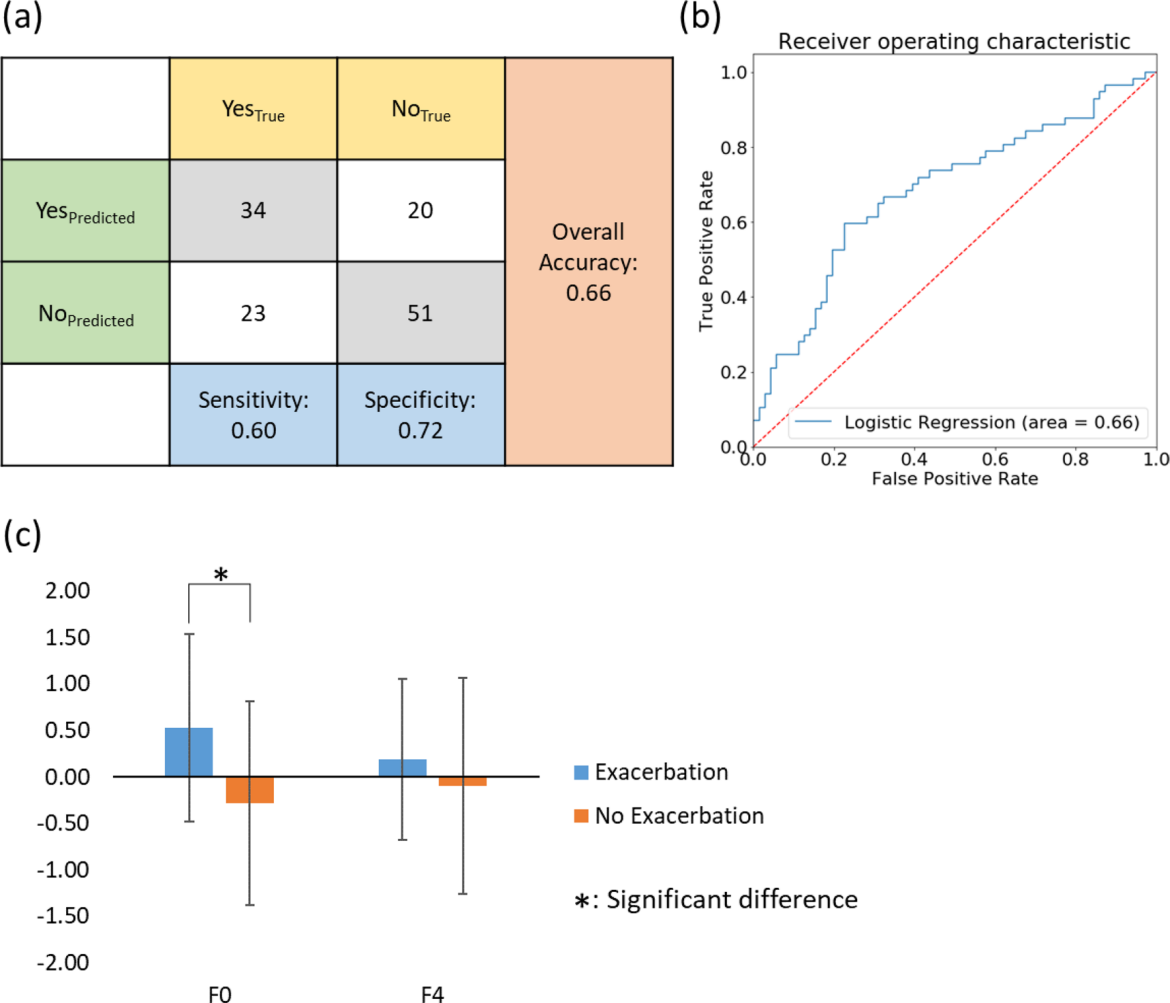
(**a**) A confusion matrix of the logistic regression model fitted for the incident of exacerbation, showing the numbers of correctly and wrongly predicted cases. (**b**) The ROC curve for the logistic regression model. (**c**) Subjects who experienced exacerbation were significantly different than those who did not in terms of F0.

Due to the fact that F0 was able to differentiate healthy non-smokers from mild COPD subjects and F4 was able to differentiate severe subjects from very severe subjects, we hypothesized that F0 and F4 could serve as predictors for exacerbation. To test this hypothesis, we developed a logistic regression model using F0 and F4 as the independent variables and the incident of exacerbation as the dependent variable. On the test dataset, an overall accuracy of 0.66, a sensitivity of 0.60, and a specificity of 0.72 were achieved for the logistic regression (Fig. 7a). The Receiver Operating Characteristic (ROC) curve with area under the curve (AUC) of 0.66 demonstrated the performance of the predictive model (Fig. 7b). In comparison, the AUC values of predictive models for exacerbation outcomes developed in previous studies varied from 0.58 to 0.81^37^. However, those models were trained based on pre-defined variables. This demonstrated the ability of our CAE-FC framework to discover meaningful latent traits purely from images without subjective feature selection. In addition, we conducted Welch’s ANOVA for comparison of subjects with and without exacerbation(s) in terms of F0 and F4. The results showed a significant difference between subjects with exacerbation(s) and those without exacerbation on F0 scores (Fig. 7c), suggesting that F0 is likely to be an indicator of lung function decline in early stages of COPD.

There are several limitations in this study. First, factors other than F0 and F4 would need further investigation since they might represent latent traits among COPD patients that have yet to be defined. For example, the representative subject of F1 shows features of combined pulmonary fibrosis and emphysema (CPFE), which is characterized by upper-lobe emphysema and lower-lobe fibrosis (Figure E6). Second, this is a cross-sectional study demonstrating the feasibility of the proposed CAE-FC and EFA framework. To confirm the relationship of cause and effect between the factors and the clinical outcomes, a longitudinal study would be required.

In this study, a CAE-FC model which automatically constructs imaging features using full 3D CT images was developed and seven factors were identified. The seven factors were significantly correlated with clinical characteristics and imaging-based variables. Two of these factors were sufficient to identify healthy non-smokers from all the subjects and differentiate COPD patients at all levels of spirometry-staged severity. Since these factors could detect abnormal tissue patterns, they could potentially serve as surrogate markers for new COPD phenotypes in detecting early abnormalities in susceptible subjects at risk and in assessing rates of lung function decline in severe patients. We hope that the recognition and interpretation of these phenotypes could eventually provide a guidance to personalized and effective care for COPD patients and improve their quality of life.

## Methods

### Ethics statement

All experiments were conducted according to relevant guidelines and regulations. The study protocols were approved by the individual institutional review boards (IRBs) of all participating institutions (Columbia University IRB 2, University of Iowa IRB-01, Johns Hopkins IRB-5, University of California Los Angeles Medical IRB 1, University of Michigan IRBMED B1 Board, National Jewish Health IRB, University of California San Francisco IRB Parnassus Panel, Temple University IRB A2, University of Alabama at Birmingham IRB #2, University of Illinois IRB #3, University of Utah IRB Panel Review Board 5, Wake Forest University IRB #5, and University of North Carolina at Chapel Hill Non-Biomedical IRB). All participants understood the purpose of the study and provided informed consent before any research activities were performed.

### Human subject data and image processing

A total of 541 former smokers and 59 healthy non-smokers (Stratum 1) were randomly selected from the SPIROMICS participants for cross-sectional analysis (Table 1). Stratum 2 represents former smokers with post-bronchodilator FEV_1_/FVC > 0.7, stratum 3 indicates those smokers with post-bronchodilator FEV_1_/FVC < 0.7 and FEV_1_% predicted > 50%, and stratum 4 indicates those smokers with FEV_1_/FVC < 0.7 and FEV_1_% predicted < 50%^38^. CT scans at both TLC and RV were acquired from multiple imaging centers^39^. All CT scans were obtained after the administration of a short-acting beta agonist bronchodilator. The former smokers were randomly split into a training dataset (containing 406 subjects) and a testing dataset (containing 135 subjects). Haghighi et al. addressed that smoking status is a confounding factor which may increase the CT density and cause the underestimation of fSAD% and Emph%^8^. Thus, this study focused on former smokers with the healthy non-smokers as a contrast group.

We registered TLC and RV images by a mass-preserving symmetric registration method^40,41^ to calculate the determinant of the Jacobian matrix for each voxel in TLC images to measure local volume change. The Jacobian accounts for the deformation of the lung from TLC to RV and allows the deep learning model to learn the expiration CT images implicitly. The Jacobian values were added as the second color channel of the TLC images. As a result, all of the imaging-based functional variables, such as PRM’s metrics, are implicitly included in the model input data. We next resampled the TLC images so that the spacing of voxels was 0.625 mm in each direction and then rescaled the CT intensities with a range from a minimum value of zero (air) to a maximum value of one (tissue) to account for the inter-site scanner difference^42,43^. The intensity of air was determined by the median (50th percentile) intensity of the trachea, and the intensity of tissue was determined by the median (50th percentile) intensity of the descending aorta. The same range of rescaled CT intensity as Jacobian in the second data channel would also make the gradient-based optimization process more efficient and stable. In addition, the PRM’s metrics were calculated with rescaled CT intensity, also known as fraction-based measures as opposed to density threshold-based measures^7,8,42^. Subsequently, lung segmentations were performed to remove the background and central airways (i.e. trachea, main bronchi) from the images. In total, 290,466 3D ROIs of 32 voxels in each direction (a total of 32^3^ voxels per ROI) were randomly extracted from all the TLC images in the training dataset. Use of random extraction instead of a sliding window approach was to prevent the model from learning the sequence of sampling the ROIs. The size of the ROI was chosen to approximate the size of a secondary lobule (20^3^ mm^3^) since the secondary pulmonary lobule is a fundamental unit of lung structure^44^. The lung and airway segmentations were processed by Apollo software (VIDA Diagnostics, Coralville, Iowa). All the image processing was done using Insight Toolkit (ITK, version 5.0).

### Convolutional autoencoder and feature constructor

Each voxel in the extracted ROIs consisted of two input channels: rescaled CT intensity and Jacobian. The ROIs were fed into a 3D CAE-FC model to learn their 1D representations. The 3D CAE-FC model contained an encoder, an embedding layer, a decoder and a FC (Fig. 1a). The encoder consisted of 3D convolutional layers, which down-sampled a 3D ROI to its 1D representation (embedding). The decoder consisted of 3D de-convolutional layers, which up-sampled the output of the FC to the original ROI. The FC comprised the six layers (Figure E1), which input the embeddings from the encoder and output the activation-regulated embeddings to the decoder. The role of the FC is to group the ROIs with similar patterns and assign a cluster number to each ROI. The FC suppressed the small activations in the embeddings and forced the decoder to reconstruct the ROIs using only important activations. That is, the ROIs were grouped based on the greatest activations among the highest-level feature maps (embeddings). Thus, the goal of training our 3D CAE-FC model is not only extracting 1D representations of the ROIs, but also grouping the 1D representations. To evaluate the efficacy of the FC to differentiate the pattern-clusters, we tested the CAE-FC model with the MNIST dataset (Figure E2, and Table E1) and compared its performance with that of k-means clustering technique (Table E2).

After model training, a sliding window technique with overlapping of 16 voxels in each direction was applied to extract ROIs, feed them to the model for cluster assignment, quantify the frequency of each pattern-cluster within the whole lung and construct a pattern-cluster histogram for each subject (Fig. 1b). We further used EFA^45–47^, a data-reduction technique, to extract factors from these pattern-clusters so that strongly correlated pattern-clusters could be grouped together into the same factor due to a similar latent trait. The number of factors were determined by parallel analysis^45^, which selects as many factors as possible as long as its eigenvalue is larger than that of random data. In addition, the optimal number of pattern-clusters was determined by the accuracy of PFT measures by using the corresponding factors while preventing redundancy. The AIC was used to select the most parsimonious model.

To demonstrate the usefulness of the factors identified, we tested their capability to predict FEV_1_% predicted and FEV_1_/FVC by constructing multivariable regression models, and compared them with those of pattern-clusters and imaging-based variables^7,8^. We used four imaging-based metrics including Emph%^8^, fSAD%^8^, the median Jacobian of the total lung, and ADI of the total lung^48^. These imaging-based variables were shown to associate with pulmonary function decline in COPD patients^49^. Moreover, to illustrate the importance and uniqueness of the factors, multivariable regression models based on factors, Emph%, and fSAD% were built and their ability in predicting pulmonary function was examined. The forward feature selection method was conducted to determine the number of predictors needed to explain sufficient variation of dependent variables in a multivariable regression model. The F-test was used to test if R^2^ changed significantly as the number of independent variables increased^50^. The process of feature selection would stop if the change of R^2^ was not significant (p > 0.05). To show the distinctions of factor scores among subgroups of different spirometry-staged severities, the mean factor scores between the healthy non-smokers and GOLD 0–4 subjects were compared using Welch’s ANOVA and the Games–Howell method for post-hoc pairwise comparisons with the significance level (α) set at 0.05.

### Exploratory factor analysis and factor interpretation

It is common to observe various forms of imaging features, but they all may be closely associated with an underlying trait. For instance, emphysema subtypes exhibit distinct texture features on CT images, but they are all associated with the latent trait of reduced lung function. In this study, EFA was applied to reduce a large number of deep-learning-based observed variables (pattern-clusters) to a lower number of latent traits (factors), aiming to model the interrelationships among pattern-clusters with factors. EFA consists of three steps: factor extraction, factor rotation, and factor Interpretation. Detailed information of factor extraction and factor rotation are addressed in supplementary material.

Based on pattern matrix coefficients which reflect the unique contribution of the pattern-clusters to the factors, the CAE-derived pattern-clusters with high factor loadings (i.e. greater than 0.5) were selected for analysis. The means and standard deviations of the averaged intensities and the averaged Jacobians of the ROIs belonging to each factor were compared Welch’s ANOVA with the Games–Howell method for post-hoc pairwise tests. In addition, to facilitate the interpretation of the identified factors, we aimed to look for those factors that are closely related to the existing known variables, such as clinical variables, imaging-based variables, biomarkers, and medication use, which have been selected and measured by SPIROMICS and Haghighi et al.^7,8^. In addition to those known variables, AWV%^26,51^, RV/TLC^30^, and airway variants (Figure E3) were also extracted from CT images for analysis. AWV% is a measure of dysanapsis and is inversely correlated with RV/TLC^51^. Dysanapsis is a risk factor for accelerated decline of lung function in COPD^26^. Genetically-determined central airway variation, which alters distal lung structure, is also a susceptibility factor for COPD^36^. Pearson’s correlation and bi-serial correlation were used to examine the associations of the identified factors with the continuous variables and binary variables, respectively. The statistical analyses were conducted using SciPy 1.4.1 and Pingouin 0.3.4 in Python 3 packages.

### Ethics approval consent to participate

Ethics and consent were approved by SPIROMICS committee.

### Consent for publication

The paper was approved by SPIROMICS Publications and Presentation Committee.

## Supplementary Information

Supplementary Information.
