# Supplementary material for: Latent traits of lung tissue patterns in former smokers derived by dual channel deep learning in computed tomography images
Source: Sci Rep. 2021 Mar 1;11:4916. doi: 10.1038/s41598-021-84547-5 (PMC7921389; doi:10.1038/s41598-021-84547-5)
Supplement: Supplementary file 1 — Supplementary Information. [file 41598_2021_84547_MOESM1_ESM.pdf]

Supplementary Materials:

Latent Traits of Lung Tissue Patterns in Former Smokers derived by Dual Channel Deep Learning in Computed Tomography Images

Frank Li, Jiwoong Choi, Chunrui Zou, John D. Newell Jr, Alejandro P. Comellas, Chang Hyun Lee, Hongseok Ko, R. Graham Barr, Eugene R. Bleecker, Christopher B. Cooper, Fereidoun Abtin, Igor Barjaktarevic, David Couper, MeiLan Han, Nadia N. Hansel, Richard E. Kanner, Robert Paine III, Ella A. Kazerooni, Fernando J. Martinez, Wanda O'Neal, Stephen I Rennard, Benjamin M. Smith, Prescott G Woodruff, Eric A. Hoffman, and Ching-Long Lin

Feature Constructor

The feature constructor consists of the following six layers (**Figure E 1**).

1. a softmax layer, taking the embedding as an input,
2. a threshold ReLU layer, suppressing the small output (i.e. value less than 1 divided by the number of pattern-clusters) of the softmax layer,
3. an amplifier, multiplying the output of the threshold ReLU layer by approximately the number of pattern-clusters,
4. a sigmoid layer, taking the output of the threshold ReLU layer as an input,
5. a threshold ReLU layer, suppressing the activations less than 0.5, and
6. a multiplication layer, multiplying the embeddings by the suppressed activations.

The embedding from the encoder was input to the FC and the activation-regulated embedding was then output to the decoder. The second threshold ReLU layer served as a classifier to group the

ROIs into different pattern-clusters. The maximum activation in the second threshold ReLU layer was chosen to be the membership of the pattern-clusters. The model was developed by using Python (version 3.6) and Tensorflow with Keras API (version 1.14.0).

Steps of Exploratory Factor Analysis

The first step is factor extraction. We used principal axis factoring (PAF)[1] as our feature extraction method. It decomposes the correlation matrix of the observed variables to obtain the eigenvalues and eigenvectors, which represent the magnitude and directions of the new feature space, respectively. PAF assumes that the total variance of the observed variables is a combination of common variance and unique variance. Common variance is the variance that is shared among the observed variables while unique variance is the variance that is not shared in common (e.g. noise). PAF is similar to principal component analysis (PCA), but it accounts for the unique variance of each variable while PCA does not.

The second step is factor rotation. The purpose of factor rotation is to obtain a simple structure for easier interpretation of the extracted factors. A simple structure means that each variable has high loadings on one factor only and each factor has high loadings for only some of the variables. Without rotation, the first factor will be the most general factor that most variables load onto and will explain the largest amount of variance, making it difficult to interpret. We used the OBLIMIN method, an oblique rotation method allowing correlations among factors, to rotate the extracted factors [1]. After factor rotation, we obtained a pattern matrix and a structure matrix. Coefficients of the pattern matrix reflect the unique factor loadings (similar to partial standardized regression coefficients), while the structure matrix contains correlations between factors and variables.

Finally, we calculated the factor scores (F_s) using **Equation (E1)** and **Equation (E2)** based on Thurstone's approach [2] for the future predictions of PFT measures or other clinical variables.

$$F_s = XW \quad \textbf{(E1)}$$

$$W = R^{-1}S = R^{-1}P\emptyset \quad \textbf{(E2)}$$

where X is the standardized pattern-histogram, W contains the regression weights, R is the correlation matrix of the pattern-histogram, S is the structure matrix, P is the pattern matrix, and $\emptyset$ is the correlation matrix of the factors. EFA was performed using R software (version 3.5.3) with the psych package (version 1.8.12).

Testing the efficacy of the feature constructor

We trained a 2D CAE-FC model with 10 embeddings, which has the same network structure with the 3D CAE-FC, using the MNIST dataset. The 2D CAE-FC model was able to group similar digits into the same pattern-clusters as show in **Figure E 2**. The percentages of each number in each cluster were shown in **Table E 1.**

Supplementary Figures

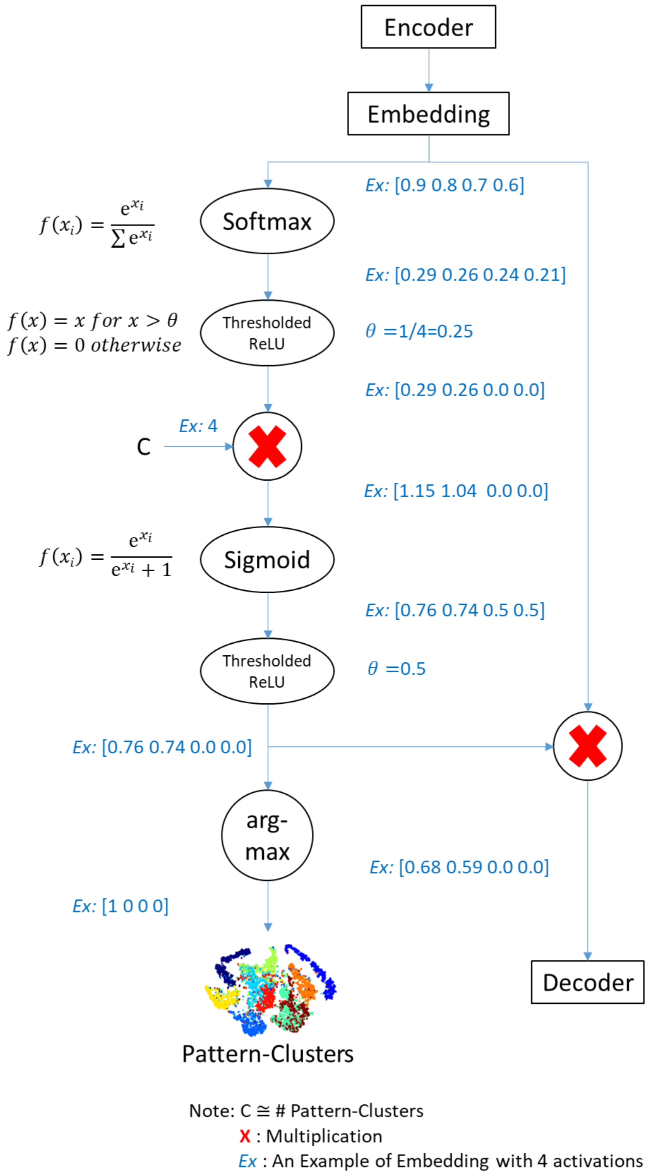

Figure E 1. The structure of the feature constructor. It takes the embedding as input and assigns the membership of pattern-cluster to each ROI. The softmax layer normalizes the embedding vector into a probability distribution between zero and one. It is considered as a soft version of the argmax function. Subsequently, the first threshold ReLU layer suppresses the activations smaller than the average probability (i.e.one divided by the number of activations). The output of the threshold ReLU layer is then multiplied by an arbitrary number (e.g. number of activations from the embedding), so that it is greater than one. Next, each activation from the last layer is normalized by the sigmoid layer and is clipped by the second threshold ReLU layer to remove the values less than or equal to 0.5. Through the steps above, we are able to obtain a vector with only important activations remaining. Lastly, the activations of the embedding are multiplied by the vector to enlarge the differences between the largest activations and other activations. The adjusted activations of the embedding are used by the decoder to reconstruct the ROIs.

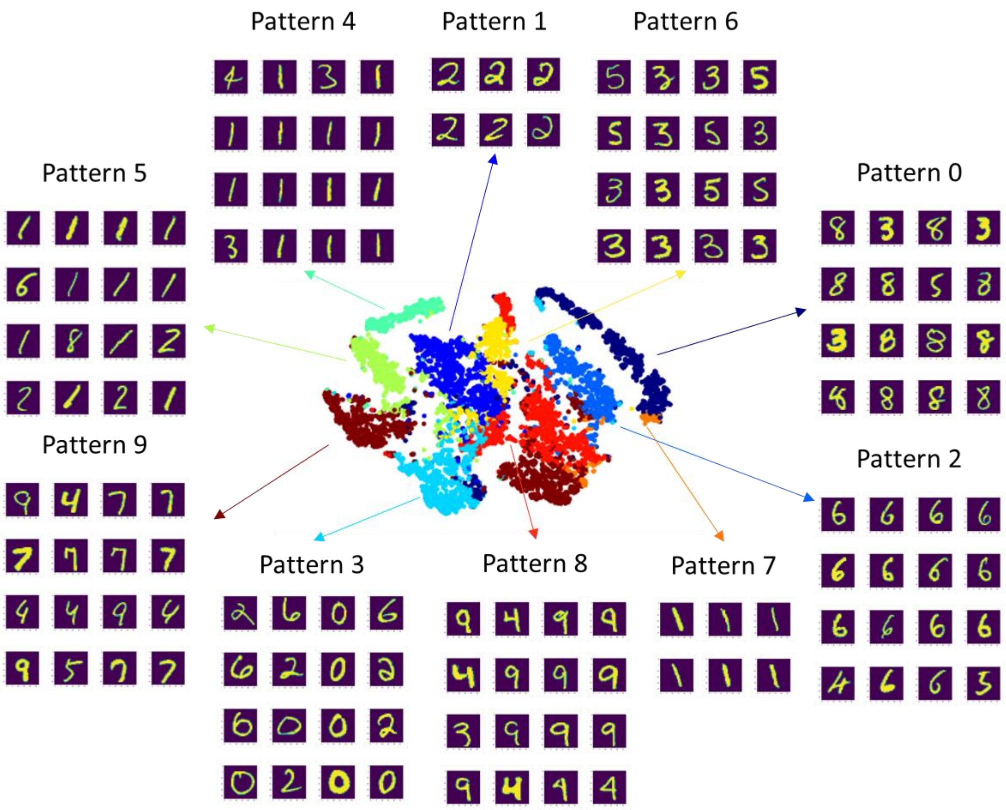

Figure E 2. Pattern-clusters identified by our CAE-FC using the MNIST dataset.

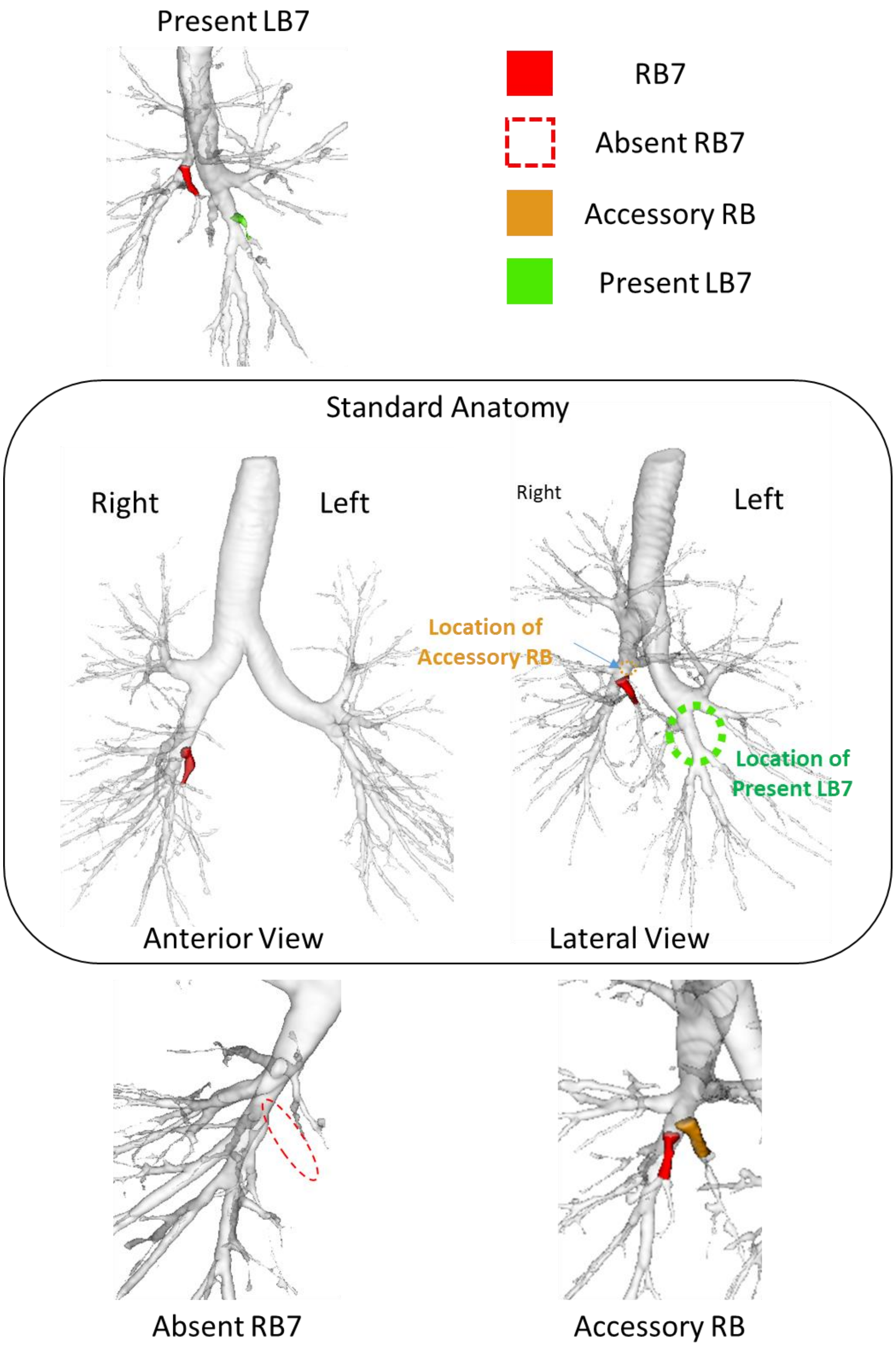

Figure E 3. Illustration of three airway variants with respect to standard airway anatomy: (1) absence of a right medial-basal RB7 branch in the right lower lobe (Absent RB7), (2) presence of an accessory sub-superior RB branch in the right lower lobe (Accessory RB), and (3) presence of an a left medial-basal LB7 in the left lower lobe (Present LB7).

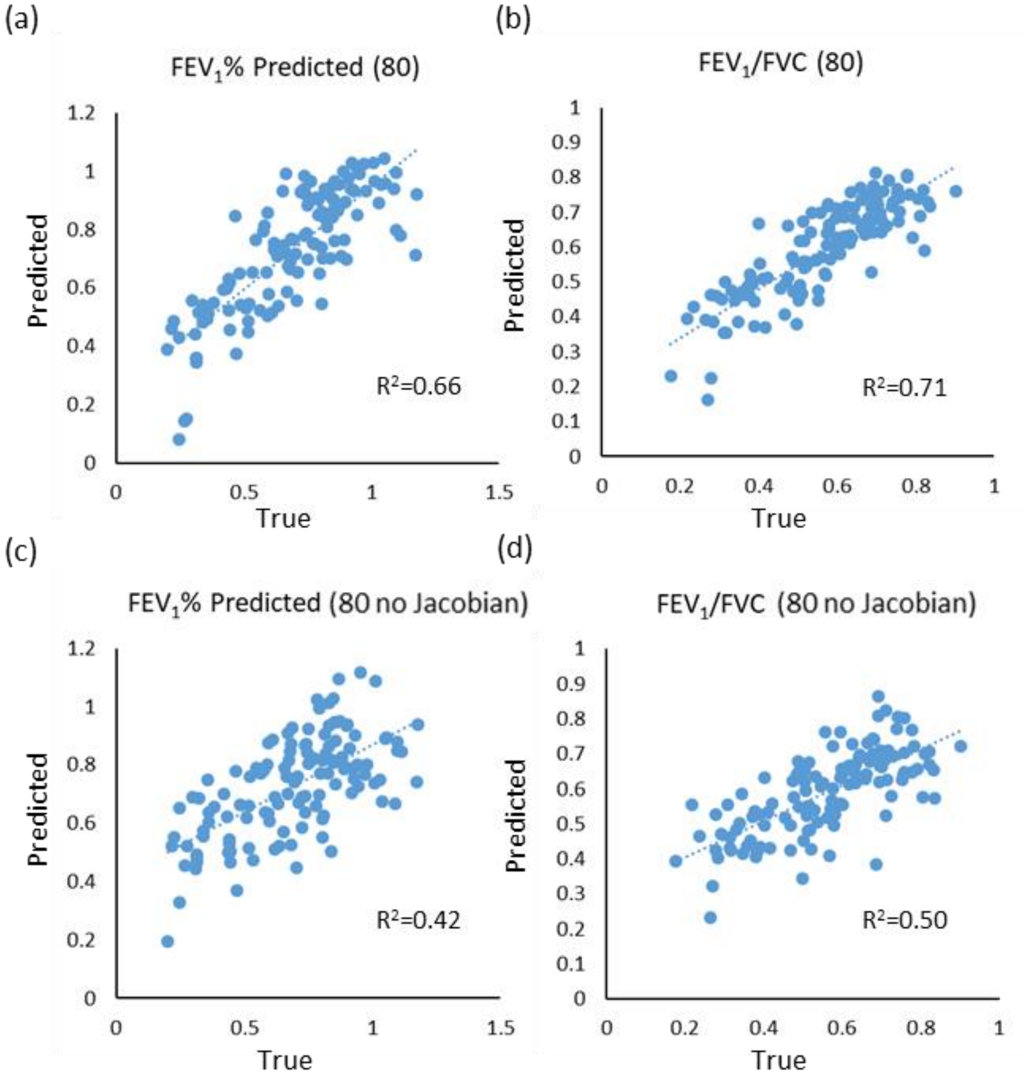

Figure E 4. The scatter plots of the true values versus the predicted values for (a) FEV_1% predicted and (b) FEV_1/FVC, using the model of seven factors extracted from 80 embeddings with Jacobian as a second channel. The scatter plots of the true values versus the predicted values for (c) FEV_1% predicted and (d) FEV_1/FVC, using the model of 80 embeddings without Jacobian as a second channel. The model with Jacobian as a second channel explained 23.9% and 20.7% more variation of FEV_1% predicted and FEV_1/FVC, respectively.

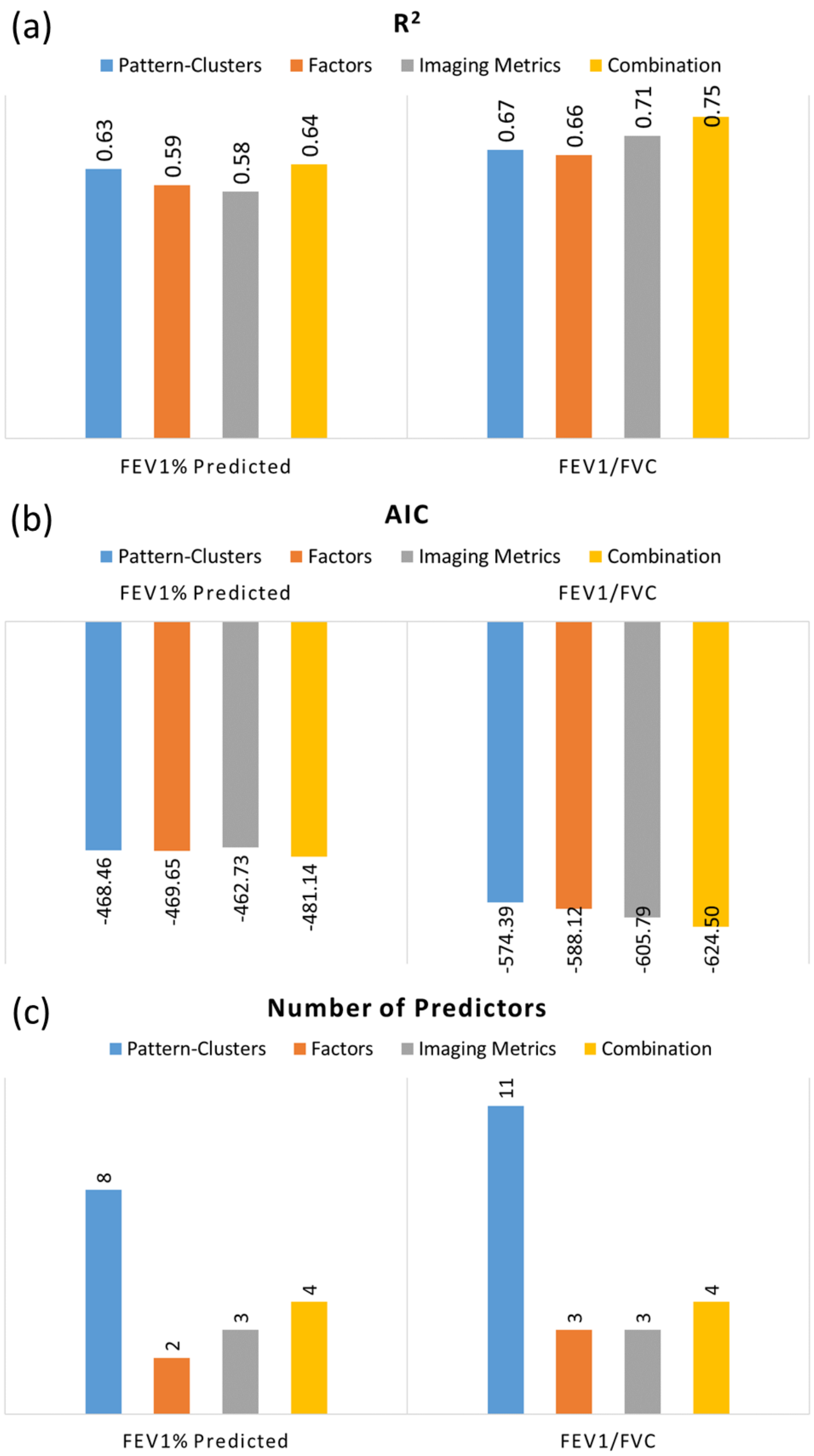

Figure E 5. Comparisons of (a) R^2, (b) AIC, and (c) number of predictors between factor-based models, pattern-cluster-based models, imaging-metric-based models, and combination models based on F0, F4, Emph% and fSAD% in regard to the ability to predict the pulmonary functions of FEV_1 and FEV_1/FVC. The combination models outperformed the imaging-metric-based models.

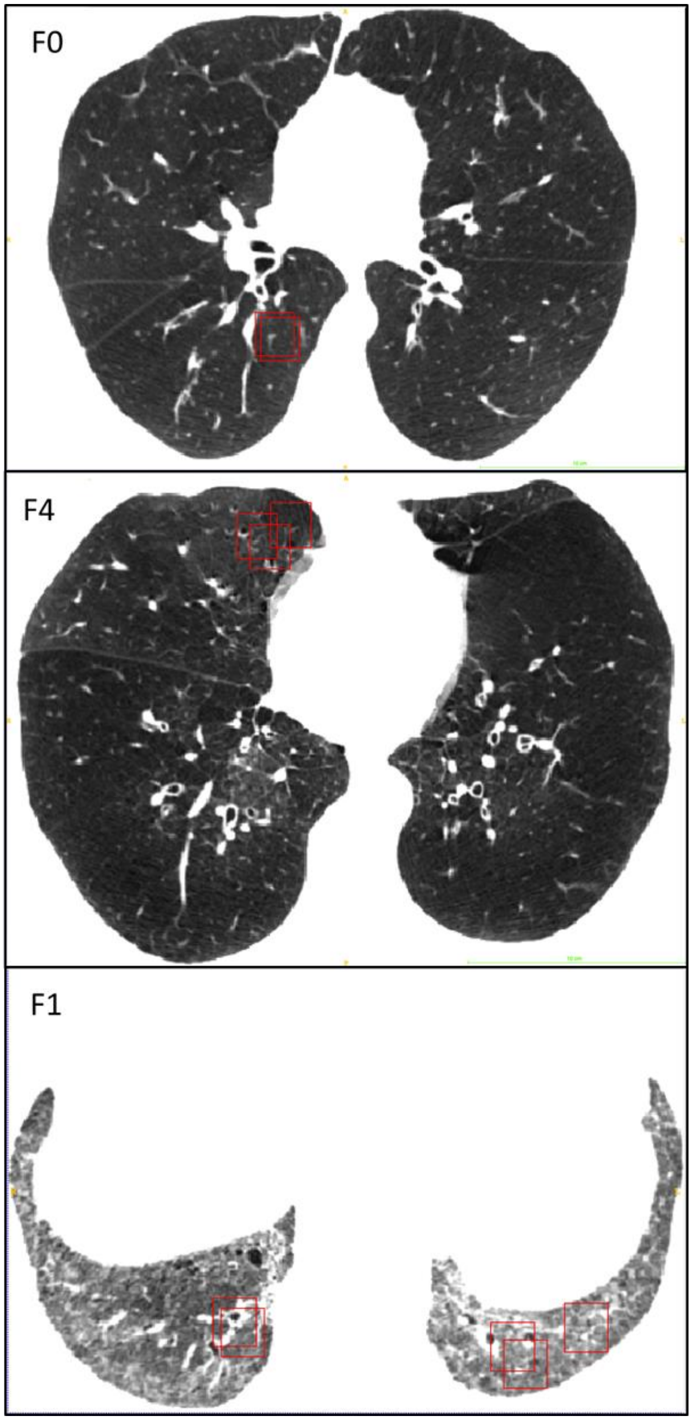

Figure E 6. Illustration of representative CT images for F0 and F4, marked with the ROIs in red bounding boxes belonging to associated pattern-clusters. A narrow window for emphysema with level of -700 HU and window of 750 HU (Lynch et al. 2015)[8] was used in these cases. A narrow window corresponds to the range of the minimum and maximum HUs to view the CT image. Using a narrow window helps radiologists identify CT features more sensitively. The ROIs for F1 appear to detect high attenuation areas in the basal lungs. These may include fibrotic features of lung with interstitial lung diseases (ILD), which is not supposedly related to smoking. CT patterns of F1 appear to be combined pulmonary fibrosis and emphysema (CPFE).

Supplementary Tables

Table E 1. The percentages (%) of each digital number in each pattern classified by the CAE-FC. The order of the columns has been rearranged so that the digit with the highest proportion in each pattern would be on the diagonal of the table. The total accuracy was 0.4440 for the CAE-FC.

	**Pattern 3**	**Pattern 4**	**Pattern 1**	**Pattern 6**	**Pattern 8**	**Pattern 7**	**Pattern 2**	**Pattern 9**	**Pattern 0**	**Pattern 5**	**Total**
**0**	36.46	0.00	2.04	1.08	0.17	0.73	1.28	0.31	4.29	3.38	
**1**	0.37	84.68	0.00	0.00	0.00	26.28	0.32	0.03	0.38	56.93	
**2**	31.85	0.23	90.48	0.54	0.67	1.46	0.00	1.49	4.52	3.04	
**3**	1.45	9.76	4.08	55.14	0.17	0.73	1.60	3.08	21.93	15.71	
**4**	1.08	1.36	0.68	0.00	49.41	0.00	5.75	21.66	0.23	0.68	
**5**	0.91	0.57	2.04	34.46	0.84	67.15	1.60	8.77	17.02	5.07	
**6**	25.66	1.02	0.68	1.35	0.51	2.19	88.50	0.14	1.30	2.70	
**7**	0.83	1.02	0.00	0.00	3.71	0.00	0.00	33.55	0.08	1.35	
**8**	1.00	0.34	0.00	6.62	0.17	1.46	0.64	6.52	49.23	10.64	
**9**	0.37	1.02	0.00	0.81	44.35	0.00	0.32	24.44	1.00	0.51	
**n**	2408	881	147	740	593	137	313	2885	1304	592	10000
**Correct**	878	746	133	408	293	92	277	968	642	3	4440

Table E 2. The percentages (%) of each digital number in each pattern classified by the k-means. The order of the columns has been rearranged so that the digit with the highest proportion in each pattern would be on the diagonal of the table. The total accuracy was 0.3642 for k-means. This result suggested that the CAE-FC model (accuracy: 0.4440) can better cluster similar patterns together than the k-means approach.

	**Pattern 3**	**Pattern 8**	**Pattern 9**	**Pattern 1**	**Pattern 4**	**Pattern 6**	**Pattern 7**	**Pattern 5**	**Pattern 2**	**Pattern 0**	**Total**
**0**	84.14	0.64	0.23	1.94	0.08	22.82	23.05	0.00	0.33	0.33	
**1**	0.00	43.20	0.45	0.97	4.29	2.54	3.91	0.00	17.63	0.00	
**2**	3.11	0.69	92.05	45.84	3.83	0.30	23.70	3.24	0.86	0.20	
**3**	1.09	25.83	1.82	1.80	10.60	5.68	3.39	8.65	17.24	1.27	
**4**	0.31	2.08	0.00	2.91	11.88	2.84	2.21	3.78	14.21	32.80	
**5**	0.16	10.71	0.23	1.11	2.78	39.86	2.73	0.54	8.55	7.63	
**6**	9.80	2.61	0.68	36.98	0.98	21.10	38.41	0.00	3.75	0.47	
**7**	0.00	0.06	0.91	1.94	42.78	0.00	0.13	70.27	1.32	10.64	
**8**	0.93	11.81	3.64	5.12	12.93	3.85	2.21	9.73	19.14	10.51	
**9**	0.47	2.37	0.00	1.39	9.85	1.01	0.26	3.78	16.97	36.14	
**n**	643	1727	440	722	1330	986	768	370	1520	1494	10000
**Correct**	541	746	405	13	158	393	295	260	291	540	3642

Table E 3. AICs for models based on different numbers of embeddings and their corresponding numbers of factors to predict FEV_1 % predicted and FEV_1/FVC.

$\mathbf{N_{Embedding}}$	$\mathbf{N_{Factor}}$	$\mathbf{AIC_{FEV_1\%predicted}}$	$\mathbf{AIC_{FEV_1/FVC}}$
16	3	-447.34	-561.75
32	6	-425.61	-545.88
48	9	-439.89	-542.34
64	8	-456.42	-561.98
80	7	-476.02	-590.98
96	8	-426.68	-531.38
112	8	-451.41	-557.53
80<br>(no Jacobian)	3	-417.69	-531.25

Table E 4. Comparison of mean factor scores for F0 - F6 using Welch's ANOVA. There were significant differences between healthy non-smokers and GOLD 0-4 subjects for F0, F2, F4, F5, and F6. Cohen's d was derived from partial η^2.

	**Mean Factor Score**	**p value**	**Partial η^2**	**Cohen's d**
**F0**	101.252	6.619E-53	0.386	0.837
**F1**	0.506	7.719E-01	0.005	0.009
**F2**	8.463	2.903E-07	0.055	0.110
**F3**	2.207	5.500E-02	0.021	0.043
**F4**	37.492	5.283E-27	0.205	0.419
**F5**	8.323	3.363E-07	0.006	0.012
**F6**	15.544	6.127E-13	0.039	0.078

Table E 5. Comparisons of mean factor scores of F0 between a pair of severity subgroups using the Games-Howell method for post-hoc pairwise tests. A and B represent the pair in the pair-wise comparisons. F0 is able to differentiate between GOLD 0 subjects and healthy non-smokers.

A	B	mean(A)	mean(B)	std.(A)	std.(B)	*p* value	Hedges' g
G0	G1	-0.742	-0.303	1.040	0.671	0.001	-0.523
G0	G2	-0.742	0.285	1.040	0.898	0.001	-1.053
G0	G3	-0.742	0.876	1.040	0.897	0.001	-1.692
G0	G4	-0.742	0.998	1.040	0.859	0.001	-1.930
G0	healthy	-0.742	-1.066	1.040	0.436	0.020	0.486
G1	G2	-0.303	0.285	0.671	0.898	0.001	-0.764
G1	G3	-0.303	0.876	0.671	0.897	0.001	-1.476
G1	G4	-0.303	0.998	0.671	0.859	0.001	-1.595
G1	healthy	-0.303	-1.066	0.671	0.436	0.001	1.414
G2	G3	0.285	0.876	0.898	0.897	0.001	-0.657
G2	G4	0.285	0.998	0.898	0.859	0.001	-0.819
G2	healthy	0.285	-1.066	0.898	0.436	0.001	2.237
G3	G4	0.876	0.998	0.897	0.859	0.900	-0.139
G3	healthy	0.876	-1.066	0.897	0.436	0.001	2.937
G4	healthy	0.998	-1.066	0.859	0.436	0.001	2.822

Table E 6. Comparisons of mean factor scores of F4 between a pair of severity subgroups using the Games-Howell method for post-hoc pairwise tests. A and B represent the pair in the pair-wise comparisons. F4 is able to differentiate the difference between GOLD 3 subjects and GOLD 4 subjects.

A	B	mean(A)	mean(B)	std.(A)	std.(B)	*p* value	Hedges' g
G0	G1	-0.410	-0.459	1.782	0.698	0.900	0.040
G0	G2	-0.410	-0.210	1.782	0.740	0.838	-0.146
G0	G3	-0.410	0.723	1.782	1.055	0.001	-0.821
G0	G4	-0.410	1.546	1.782	1.119	0.001	-1.524
G0	healthy	-0.410	-0.383	1.782	0.349	0.900	-0.027
G1	G2	-0.459	-0.210	0.698	0.740	0.080	-0.348
G1	G3	-0.459	0.723	0.698	1.055	0.001	-1.306
G1	G4	-0.459	1.546	0.698	1.119	0.001	-1.955
G1	healthy	-0.459	-0.383	0.698	0.349	0.900	-0.149
G2	G3	-0.210	0.723	0.740	1.055	0.001	-0.978
G2	G4	-0.210	1.546	0.740	1.119	0.001	-1.657
G2	healthy	-0.210	-0.383	0.740	0.349	0.196	0.351
G3	G4	0.723	1.546	1.055	1.119	0.002	-0.744
G3	healthy	0.723	-0.383	1.055	0.349	0.001	1.533
G4	healthy	1.546	-0.383	1.119	0.349	0.001	2.118

Table E 7. The predictors of PFTs chosen by the forward feature selection method for the factor-based model, the imaging-metric-based models, and the combination models.

	**Predictors**		
	**Factor-based models**	**Imaging-metric-based models**	**Combination models**
**FEV_1% predicted**	F0, F4	Emph%, ADI, fSAD%	Emph%, fSAD%, F0, F4
**FEV_1/FVC**	F0, F4, F5	Emph%, fSAD%, Jacobian	Emph%, fSAD%, F0, F4

Table E 8. Comparisons of averaged CT intensity (i.e. first channel of ROIs) and averaged Jacobian (i.e. second channel of ROIs) between each factor using the Games-Howell method for post-hoc pairwise tests. A and B represent the pair in the pair-wise comparisons.

A	B	CT Intensity					Jacobian				
		mean(A)	std(A)	mean(B)	std(B)	p	mean(A)	std(A)	mean(B)	std(B)	p
F0	F1	0.067	0.030	0.256	0.078	<0.001	0.664	0.088	0.784	0.091	<0.001
F0	F2	0.067	0.030	0.135	0.042	<0.001	0.664	0.088	0.741	0.074	<0.001
F0	F3	0.067	0.030	0.194	0.059	<0.001	0.664	0.088	0.696	0.106	0.007
F0	F4	0.067	0.030	0.062	0.035	<0.001	0.664	0.088	0.914	0.067	<0.001
F0	F5	0.067	0.030	0.113	0.023	<0.001	0.664	0.088	0.540	0.061	<0.001
F0	F6	0.067	0.030	0.107	0.035	<0.001	0.664	0.088	0.513	0.081	<0.001
F1	F2	0.256	0.078	0.135	0.042	<0.001	0.784	0.091	0.741	0.074	<0.001
F1	F3	0.256	0.078	0.194	0.059	<0.001	0.784	0.091	0.696	0.106	<0.001
F1	F4	0.256	0.078	0.062	0.035	<0.001	0.784	0.091	0.914	0.067	<0.001
F1	F5	0.256	0.078	0.113	0.023	<0.001	0.784	0.091	0.540	0.061	<0.001
F1	F6	0.256	0.078	0.107	0.035	<0.001	0.784	0.091	0.513	0.081	<0.001
F2	F3	0.135	0.042	0.194	0.059	<0.001	0.741	0.074	0.696	0.106	<0.001
F2	F4	0.135	0.042	0.062	0.035	<0.001	0.741	0.074	0.914	0.067	<0.001
F2	F5	0.135	0.042	0.113	0.023	<0.001	0.741	0.074	0.540	0.061	<0.001
F2	F6	0.135	0.042	0.107	0.035	<0.001	0.741	0.074	0.513	0.081	<0.001
F3	F4	0.194	0.059	0.062	0.035	<0.001	0.696	0.106	0.914	0.067	<0.001
F3	F5	0.194	0.059	0.113	0.023	<0.001	0.696	0.106	0.540	0.061	<0.001
F3	F6	0.194	0.059	0.107	0.035	<0.001	0.696	0.106	0.513	0.081	<0.001
F4	F5	0.062	0.035	0.113	0.023	<0.001	0.914	0.067	0.540	0.061	<0.001
F4	F6	0.062	0.035	0.107	0.035	<0.001	0.914	0.067	0.513	0.081	<0.001
F5	F6	0.113	0.023	0.107	0.035	0.718	0.540	0.061	0.513	0.081	0.118

Table E 9. The percentages of standard airway anatomy and variants among the former smokers in this study, compared with those reported by Smith et al. [9]. Since airway variants are not mutually exclusive, the sum of the percentages may exceed unity.

	**Smith et al., 2018**	**Current Study**
**Standard anatomy**	73.50%	61.52%
**Accessory sub-superior**		
**Accessory RB**	16.00%	14.63%
**Accessory LB**		6.41%
**Medial-basal**		
**Absent RB7**	6.10%	11.22%
**Present LB7**	< 4.00%	11.62%

Table E 10. The correlations between each of the seven factors and related clinical variables, imaging-based variables, and use of drugs. See **Table E 11** for the descriptions of the variables.

	F0		F1		F2		F3		F4		F5		F6	
	**r**	**p**	**r**	**p**	**r**	**p**	**r**	**p**	**r**	**p**	**r**	**p**	**r**	**p**
**ADVAIR**	0.052	4.90E-01	0.031	6.77E-01	-0.071	3.45E-01	-0.009	9.02E-01	0.008	9.12E-01	-0.003	9.63E-01	0.106	1.57E-01
**AEROBID**	0.019	8.00E-01	-0.041	5.88E-01	0.189	1.29E-02	0.072	3.47E-01	-0.028	7.14E-01	-0.027	7.24E-01	-0.075	3.29E-01
**AGE_DERV_01**	0.070	1.13E-01	-0.007	8.82E-01	0.071	1.07E-01	0.002	9.61E-01	-0.005	9.16E-01	0.125	4.31E-03	0.051	2.44E-01
**ALBUTEROL**	-0.006	9.26E-01	-0.035	5.92E-01	0.102	1.12E-01	0.045	4.86E-01	-0.020	7.57E-01	0.023	7.25E-01	-0.127	4.88E-02
**ALBUTEROL_NEB**	-0.316	2.89E-02	-0.061	6.80E-01	-0.020	8.92E-01	-0.178	2.26E-01	-0.132	3.71E-01	0.284	5.05E-02	0.226	1.23E-01
**ALBUTEROLIPRATROPIUMBROMIDE**	0.223	1.24E-01	0.193	1.84E-01	0.162	2.66E-01	0.266	6.50E-02	0.356	1.21E-02	-0.326	2.24E-02	0.028	8.48E-01
**APNEA_DIAGNOSED01**	-0.086	5.53E-02	0.062	1.69E-01	-0.086	5.37E-02	0.076	8.83E-02	-0.030	5.08E-01	0.058	1.99E-01	0.111	1.33E-02
**ARFORMOTEROL**	0.293	4.32E-02	0.062	6.73E-01	0.053	7.21E-01	0.264	6.98E-02	0.039	7.92E-01	-0.160	2.79E-01	-0.107	4.68E-01
**AZMACORT**	-0.161	3.54E-02	0.358	1.39E-06	0.107	1.61E-01	-0.011	8.90E-01	-0.018	8.17E-01	0.165	3.01E-02	0.060	4.34E-01
**BMH08B**	0.059	1.84E-01	-0.014	7.56E-01	0.058	1.92E-01	0.009	8.47E-01	-0.031	4.88E-01	0.053	2.33E-01	-0.021	6.42E-01
**BMH08D**	-0.002	9.72E-01	-0.004	9.32E-01	-0.004	9.37E-01	-0.006	8.98E-01	0.002	9.65E-01	-0.020	6.56E-01	0.021	6.34E-01
**BMH08H**	-0.036	4.09E-01	0.103	1.91E-02	0.029	5.08E-01	0.021	6.37E-01	0.027	5.42E-01	0.091	3.85E-02	-0.030	5.01E-01
**BMH13A**	-0.086	5.06E-02	0.135	2.19E-03	0.038	3.93E-01	0.074	9.37E-02	-0.033	4.62E-01	0.110	1.29E-02	0.031	4.78E-01
**BMI_CM01**	-0.161	2.38E-04	0.211	1.21E-06	-0.109	1.31E-02	0.057	1.97E-01	-0.078	7.65E-02	0.275	1.97E-10	0.055	2.15E-01
**BUDESONIDEFORMOTEROL**	0.123	6.18E-02	-0.105	1.12E-01	0.032	6.32E-01	0.078	2.39E-01	0.090	1.72E-01	-0.120	6.90E-02	-0.150	2.21E-02
**CB_DIAGNOSED01**	0.138	2.03E-03	0.116	9.52E-03	-0.001	9.89E-01	0.117	9.25E-03	0.139	1.86E-03	-0.020	6.50E-01	-0.034	4.52E-01
**CB_VISIT1**	0.029	5.19E-01	0.063	1.55E-01	0.005	9.10E-01	0.031	4.84E-01	0.086	5.46E-02	0.009	8.41E-01	-0.031	4.93E-01
**CBC_BASOPHIL_PCT01**	0.007	8.72E-01	-0.054	2.26E-01	-0.097	2.89E-02	-0.022	6.17E-01	-0.039	3.77E-01	0.011	7.99E-01	0.045	3.12E-01
**CBC_EOSINOPHIL_PCT01**	0.017	7.00E-01	0.004	9.30E-01	-0.066	1.34E-01	0.050	2.55E-01	-0.018	6.91E-01	0.003	9.37E-01	-0.030	5.00E-01
**CBC_LYMPHOCYTE_PCT01**	-0.162	2.27E-04	0.010	8.18E-01	-0.011	8.06E-01	-0.061	1.66E-01	-0.123	5.23E-03	0.052	2.38E-01	-0.044	3.24E-01
**CBC_MONOCYTE_PCT01**	0.001	9.83E-01	-0.002	9.59E-01	0.027	5.36E-01	0.061	1.71E-01	-0.018	6.92E-01	-0.013	7.62E-01	0.057	1.96E-01
**CBC_NEUTROPHIL_PCT01**	0.126	4.38E-03	0.001	9.83E-01	0.021	6.36E-01	0.015	7.30E-01	0.112	1.12E-02	-0.031	4.80E-01	0.031	4.78E-01
**CBC07_DERV**	-0.011	8.13E-01	0.048	3.21E-01	0.096	4.58E-02	0.042	3.80E-01	0.019	6.92E-01	0.113	1.88E-02	0.010	8.35E-01
**CBC08_DERV**	0.164	1.97E-04	0.055	2.10E-01	0.010	8.14E-01	0.073	9.95E-02	0.135	2.24E-03	0.064	1.50E-01	-0.030	4.98E-01
**COPD_DIAGNOSED01**	0.362	4.87E-17	0.016	7.24E-01	0.085	5.57E-02	0.114	1.06E-02	0.262	2.42E-09	-0.151	6.87E-04	0.022	6.28E-01
**COPDSCORE0101**	0.212	2.45E-06	0.106	1.95E-02	0.120	7.93E-03	0.099	2.88E-02	0.315	1.18E-12	-0.009	8.45E-01	-0.029	5.17E-01
**EMPHYSEMA_DIAGNOSED01**	0.277	3.28E-10	0.057	2.04E-01	0.086	5.52E-02	0.129	3.94E-03	0.276	3.96E-10	-0.086	5.64E-02	-0.028	5.40E-01
**ETHNICITY**	-0.132	2.66E-03	0.138	1.65E-03	-0.002	9.70E-01	0.036	4.10E-01	0.073	9.78E-02	0.053	2.25E-01	-0.053	2.24E-01
**FEV1/FVC_norm**	-0.698	5.74E-77	0.039	3.76E-01	-0.168	1.25E-04	-0.101	2.08E-02	-0.475	1.31E-30	0.318	1.12E-13	0.046	2.92E-01
**FLOVENT**	0.070	3.54E-01	-0.024	7.57E-01	-0.043	5.72E-01	-0.014	8.56E-01	-0.002	9.78E-01	-0.036	6.31E-01	0.082	2.81E-01

**FLUTICASONESALMETEROL**	-0.054	4.08E-01	-0.038	5.62E-01	-0.027	6.75E-01	0.065	3.23E-01	-0.023	7.22E-01	-0.036	5.86E-01	0.031	6.31E-01
**FORMOTEROL**	0.134	4.20E-02	0.018	7.80E-01	0.010	8.80E-01	0.006	9.31E-01	-0.013	8.41E-01	-0.055	4.05E-01	0.011	8.72E-01
**FORMOTEROL_NEB**	-0.124	3.97E-01	-0.183	2.07E-01	0.111	4.50E-01	-0.094	5.22E-01	0.169	2.47E-01	-0.099	5.00E-01	-0.035	8.13E-01
**GENDER**	-0.223	3.00E-07	-0.138	1.58E-03	0.058	1.84E-01	-0.242	2.22E-08	0.119	6.59E-03	0.005	9.18E-01	-0.354	8.66E-17
**IPRATBROMIALBUTSULFA**	-0.025	7.04E-01	-0.053	4.26E-01	-0.036	5.87E-01	-0.107	1.05E-01	0.020	7.57E-01	-0.040	5.48E-01	0.089	1.78E-01
**IPRATROPIUMBROMIDE**	0.058	3.81E-01	-0.020	7.66E-01	-0.025	7.06E-01	0.041	5.33E-01	-0.019	7.68E-01	-0.064	3.29E-01	-0.059	3.69E-01
**IPRATROPIUMBROMIDE_NEB**	-0.018	9.05E-01	-0.072	6.26E-01	-0.183	2.13E-01	-0.169	2.50E-01	-0.162	2.71E-01	0.104	4.82E-01	-0.255	7.97E-02
**LEVALBUTEROL**	0.030	6.48E-01	-0.007	9.11E-01	-0.028	6.68E-01	0.043	5.13E-01	-0.068	3.01E-01	-0.023	7.32E-01	-0.007	9.12E-01
**METAPROTERENOL**	0.079	2.33E-01	0.059	3.73E-01	0.206	1.59E-03	0.140	3.26E-02	0.116	7.80E-02	-0.042	5.23E-01	0.017	7.94E-01
**OTHERSTEROIDS**	0.037	6.35E-01	-0.068	3.84E-01	0.079	3.10E-01	-0.032	6.76E-01	0.016	8.37E-01	-0.003	9.67E-01	-0.140	6.99E-02
**PCT_POST_FEV1_V1_norm**	-0.647	8.13E-63	-0.037	4.06E-01	-0.249	9.22E-09	-0.134	2.24E-03	-0.510	1.22E-35	0.169	1.05E-04	0.128	3.42E-03
**PEX_SEVERE**	0.196	6.97E-06	0.064	1.46E-01	0.125	4.42E-03	0.159	2.67E-04	0.200	4.61E-06	-0.057	1.95E-01	-0.060	1.70E-01
**PEX_TOT**	0.262	1.32E-09	0.034	4.42E-01	0.105	1.67E-02	0.170	9.63E-05	0.251	6.96E-09	-0.093	3.49E-02	-0.080	6.84E-02
**PEX_TOT0101**	0.158	3.14E-04	-0.038	3.89E-01	0.106	1.55E-02	0.038	3.89E-01	0.253	5.15E-09	-0.105	1.69E-02	-0.133	2.33E-03
**PIRBUTEROL**	0.111	9.14E-02	-0.012	8.54E-01	-0.051	4.37E-01	-0.036	5.81E-01	0.017	7.98E-01	-0.040	5.46E-01	0.026	6.96E-01
**POSBD_FEV1**	-0.477	8.93E-31	0.030	4.90E-01	-0.287	2.49E-11	-0.020	6.41E-01	-0.539	2.26E-40	0.126	4.17E-03	0.336	3.84E-15
**POSBD_FVC**	-0.049	2.69E-01	0.019	6.71E-01	-0.288	2.38E-11	0.058	1.88E-01	-0.392	1.58E-20	-0.085	5.29E-02	0.465	3.32E-29
**PULMICORT**	-0.022	7.73E-01	-0.063	4.12E-01	0.052	4.95E-01	-0.127	9.69E-02	-0.042	5.80E-01	-0.003	9.70E-01	-0.041	5.96E-01
**PULMONARY_VASCULAR_CONDITION01**	0.055	2.17E-01	0.048	2.80E-01	0.078	8.04E-02	0.075	8.89E-02	0.085	5.52E-02	0.004	9.30E-01	0.001	9.82E-01
**QVAR**	-0.163	3.28E-02	-0.037	6.26E-01	-0.060	4.36E-01	-0.027	7.22E-01	-0.147	5.51E-02	0.061	4.25E-01	0.033	6.63E-01
**RDS08**	0.189	1.54E-05	0.067	1.30E-01	0.085	5.27E-02	0.075	9.01E-02	0.102	2.05E-02	-0.001	9.78E-01	-0.001	9.91E-01
**RDS12**	0.034	4.45E-01	0.042	3.42E-01	0.106	1.62E-02	-0.004	9.37E-01	0.027	5.36E-01	-0.018	6.85E-01	-0.033	4.52E-01
**RDS16**	0.031	4.84E-01	-0.028	5.32E-01	0.032	4.63E-01	-0.003	9.50E-01	-0.020	6.56E-01	-0.009	8.44E-01	-0.071	1.08E-01
**RDS25A3**	0.097	2.76E-02	0.004	9.19E-01	-0.043	3.34E-01	-0.031	4.86E-01	0.012	7.78E-01	-0.018	6.86E-01	-0.036	4.14E-01
**RDS25B3**	0.109	1.33E-02	-0.010	8.25E-01	0.022	6.23E-01	0.048	2.74E-01	-0.008	8.49E-01	-0.028	5.32E-01	-0.039	3.73E-01
**RMU09A1**	-0.197	5.14E-02	0.009	9.30E-01	-0.116	2.55E-01	-0.041	6.89E-01	-0.015	8.84E-01	-0.153	1.34E-01	0.047	6.46E-01
**RMU09A10**	0.166	1.09E-01	-0.017	8.73E-01	-0.041	6.93E-01	0.059	5.74E-01	-0.198	5.59E-02	0.019	8.56E-01	-0.017	8.74E-01
**RMU09A2**	0.028	7.85E-01	-0.047	6.47E-01	0.136	1.87E-01	-0.036	7.24E-01	0.025	8.06E-01	-0.029	7.80E-01	-0.065	5.29E-01
**RMU09A3**	-0.112	2.79E-01	0.201	4.95E-02	0.015	8.86E-01	-0.010	9.24E-01	0.021	8.39E-01	0.137	1.85E-01	0.007	9.42E-01
**RMU09A5**	0.222	2.98E-02	-0.073	4.79E-01	0.000	9.98E-01	-0.004	9.70E-01	-0.045	6.66E-01	-0.086	4.07E-01	-0.118	2.53E-01
**RMU09A6**	-0.047	6.47E-01	-0.012	9.10E-01	0.026	8.01E-01	-0.007	9.43E-01	-0.009	9.29E-01	0.173	9.14E-02	0.080	4.40E-01
**RMU09A7**	0.050	6.27E-01	-0.046	6.58E-01	-0.012	9.08E-01	0.009	9.33E-01	-0.144	1.61E-01	-0.049	6.34E-01	-0.042	6.82E-01
**RMU09A8**	0.191	6.44E-02	0.025	8.10E-01	0.143	1.68E-01	-0.120	2.45E-01	0.164	1.11E-01	0.099	3.42E-01	-0.056	5.87E-01
**RMU09A9**	0.092	3.72E-01	0.007	9.44E-01	-0.033	7.51E-01	0.085	4.11E-01	-0.187	6.84E-02	-0.015	8.82E-01	0.059	5.66E-01

**RMU11**	0.023	6.11E-01	0.034	4.46E-01	0.043	3.38E-01	-0.004	9.31E-01	-0.083	6.17E-02	0.028	5.36E-01	0.124	5.48E-03
**SALMETEROL**	0.040	5.44E-01	0.004	9.56E-01	0.137	3.68E-02	0.066	3.12E-01	0.135	3.94E-02	-0.006	9.28E-01	0.048	4.61E-01
**SIX_MINUTE_WALK_DISTANCE01**	-0.214	1.32E-06	-0.047	2.90E-01	-0.140	1.62E-03	-0.038	3.91E-01	-0.406	2.81E-21	0.055	2.19E-01	0.134	2.66E-03
**SMOKING_PACK_YEARS01**	0.147	7.92E-04	0.074	9.15E-02	-0.036	4.15E-01	0.067	1.25E-01	-0.019	6.66E-01	0.036	4.18E-01	0.121	5.70E-03
**SMW_DSAT01**	0.285	9.00E-11	0.060	1.78E-01	0.092	3.94E-02	0.110	1.39E-02	0.377	2.47E-18	-0.119	7.96E-03	-0.104	1.98E-02
**SYMBICORT**	0.043	5.78E-01	-0.045	5.56E-01	0.036	6.39E-01	0.040	6.02E-01	0.056	4.67E-01	-0.050	5.19E-01	-0.063	4.12E-01
**TIOTROPIUM**	0.104	1.06E-01	-0.007	9.18E-01	0.086	1.80E-01	0.001	9.93E-01	0.156	1.48E-02	-0.064	3.24E-01	-0.027	6.72E-01
**V1_MMP3**	0.185	7.60E-05	0.029	5.42E-01	0.042	3.72E-01	0.110	1.95E-02	0.109	1.99E-02	-0.071	1.30E-01	0.084	7.48E-02
**WHEEZINGAGE**	0.034	5.99E-01	0.062	3.33E-01	0.033	6.02E-01	0.082	2.01E-01	-0.040	5.30E-01	0.068	2.90E-01	-0.044	4.87E-01
**Emphysratio_Emphys_Total**	0.544	1.44E-40	-0.061	1.70E-01	-0.052	2.37E-01	0.114	1.02E-02	0.505	2.50E-34	-0.477	2.93E-30	0.036	4.22E-01
**Emphysratio_Emphys_RUL**	0.441	1.29E-25	0.005	9.04E-01	-0.041	3.55E-01	0.156	4.21E-04	0.479	1.41E-30	-0.379	7.09E-19	0.019	6.67E-01
**Emphysratio_Emphys_RML**	0.479	1.52E-30	-0.107	1.56E-02	-0.095	3.23E-02	0.026	5.55E-01	0.355	1.45E-16	-0.429	3.12E-24	0.032	4.76E-01
**Emphysratio_Emphys_RLL**	0.520	1.19E-36	-0.133	2.68E-03	-0.025	5.73E-01	0.041	3.61E-01	0.444	5.11E-26	-0.466	7.48E-29	-0.009	8.43E-01
**Emphysratio_Emphys_LUL**	0.483	4.37E-31	-0.040	3.67E-01	-0.079	7.63E-02	0.116	8.56E-03	0.435	6.51E-25	-0.435	6.93E-25	0.041	3.54E-01
**Emphysratio_Emphys_LLL**	0.528	7.25E-38	-0.126	4.40E-03	-0.014	7.46E-01	0.037	4.03E-01	0.440	1.74E-25	-0.457	1.15E-27	0.016	7.20E-01
**fSADratio_Emphys_Total**	0.686	5.14E-72	-0.040	3.70E-01	0.203	3.87E-06	0.041	3.58E-01	0.491	3.30E-32	-0.355	1.59E-16	-0.132	2.90E-03
**fSADratio_Emphys_RUL**	0.668	3.74E-67	-0.022	6.25E-01	0.179	4.92E-05	0.062	1.63E-01	0.461	3.43E-28	-0.347	6.83E-16	-0.137	2.00E-03
**fSADratio_Emphys_RML**	0.627	5.77E-57	-0.054	2.24E-01	0.182	3.69E-05	0.007	8.77E-01	0.269	6.95E-10	-0.308	1.14E-12	-0.081	6.87E-02
**fSADratio_Emphys_RLL**	0.603	1.04E-51	-0.070	1.16E-01	0.159	3.20E-04	-0.005	9.11E-01	0.531	2.69E-38	-0.342	2.17E-15	-0.165	1.77E-04
**fSADratio_Emphys_LUL**	0.668	5.22E-67	-0.030	5.00E-01	0.206	2.72E-06	0.051	2.52E-01	0.418	5.38E-23	-0.344	1.36E-15	-0.108	1.47E-02
**fSADratio_Emphys_LLL**	0.614	4.53E-54	-0.050	2.64E-01	0.196	8.44E-06	0.024	5.94E-01	0.500	1.54E-33	-0.318	2.16E-13	-0.138	1.83E-03
**airtrapratio_AirT_Total**	0.725	9.25E-84	-0.054	2.26E-01	0.144	1.17E-03	0.076	8.98E-02	0.557	1.26E-42	-0.429	4.12E-24	-0.118	7.66E-03
**airtrapratio_AirT_RUL**	0.673	5.63E-68	-0.015	7.45E-01	0.120	7.01E-03	0.116	9.26E-03	0.544	2.58E-40	-0.402	5.12E-21	-0.122	5.93E-03
**airtrapratio_AirT_RML**	0.678	4.08E-69	-0.098	2.77E-02	0.118	8.15E-03	0.002	9.71E-01	0.362	4.11E-17	-0.411	5.32E-22	-0.105	1.77E-02
**airtrapratio_AirT_RLL**	0.650	7.09E-62	-0.091	4.10E-02	0.133	2.68E-03	0.014	7.62E-01	0.562	2.82E-43	-0.401	6.22E-21	-0.168	1.47E-04
**airtrapratio_AirT_LUL**	0.695	2.47E-74	-0.042	3.48E-01	0.137	2.04E-03	0.080	7.35E-02	0.492	2.88E-32	-0.414	1.99E-22	-0.103	2.07E-02
**airtrapratio_AirT_LLL**	0.647	2.58E-61	-0.072	1.07E-01	0.165	1.98E-04	0.033	4.52E-01	0.530	5.17E-38	-0.370	7.82E-18	-0.131	3.11E-03
**med_J_Total**	0.488	2.40E-32	0.221	3.81E-07	0.548	5.47E-42	0.086	5.12E-02	0.666	9.58E-68	0.049	2.66E-01	-0.097	2.74E-02
**med_J_RUL**	0.505	6.03E-35	0.166	1.49E-04	0.542	5.99E-41	0.072	1.03E-01	0.607	1.42E-53	0.045	3.02E-01	-0.105	1.67E-02
**med_J_RML**	0.471	5.04E-30	0.197	6.16E-06	0.510	9.54E-36	0.090	4.03E-02	0.597	2.44E-51	0.051	2.49E-01	-0.087	4.84E-02
**med_J_RLL**	0.423	6.28E-24	0.242	2.52E-08	0.482	1.72E-31	0.055	2.12E-01	0.675	2.22E-70	0.036	4.09E-01	-0.101	2.17E-02
**med_J_LUL**	0.487	3.29E-32	0.187	1.80E-05	0.551	1.82E-42	0.069	1.15E-01	0.601	2.96E-52	0.047	2.83E-01	-0.081	6.64E-02
**med_J_LLL**	0.420	1.35E-23	0.256	3.17E-09	0.513	2.94E-36	0.087	4.82E-02	0.663	4.40E-67	0.059	1.78E-01	-0.097	2.74E-02
**med_TLC_tissue_Total**	-0.559	7.34E-44	0.356	6.68E-17	0.110	1.20E-02	-0.031	4.76E-01	-0.192	1.10E-05	0.672	2.10E-69	-0.008	8.51E-01

**med_TLC_tissue_RUL**	-0.514	2.61E-36	0.220	4.31E-07	0.109	1.30E-02	-0.110	1.24E-02	-0.284	4.35E-11	0.587	2.91E-49	0.005	9.16E-01
**med_TLC_tissue_RML**	-0.457	3.86E-28	0.320	8.17E-14	0.138	1.64E-03	0.001	9.91E-01	-0.049	2.61E-01	0.539	2.40E-40	-0.034	4.39E-01
**med_TLC_tissue_RLL**	-0.489	1.95E-32	0.448	6.11E-27	0.088	4.59E-02	0.053	2.27E-01	-0.132	2.58E-03	0.648	3.93E-63	0.057	1.97E-01
**med_TLC_tissue_LUL**	-0.521	2.08E-37	0.293	9.88E-12	0.113	9.83E-03	-0.053	2.31E-01	-0.173	7.53E-05	0.615	2.92E-55	-0.023	6.00E-01
**med_TLC_tissue_LLL**	-0.494	3.17E-33	0.439	8.53E-26	0.080	6.73E-02	0.076	8.56E-02	-0.087	4.85E-02	0.633	2.69E-59	-0.011	8.09E-01
**med_ADI_Total**	-0.382	1.67E-19	-0.134	2.25E-03	-0.472	4.00E-30	0.004	9.30E-01	-0.489	1.32E-32	-0.087	4.79E-02	0.075	8.98E-02
**med_ADI_RUL**	-0.391	2.19E-20	-0.089	4.27E-02	-0.458	2.80E-28	-0.037	3.95E-01	-0.414	7.36E-23	-0.074	9.07E-02	0.019	6.65E-01
**med_ADI_RML**	-0.379	3.55E-19	-0.256	3.33E-09	-0.484	6.97E-32	-0.120	6.21E-03	-0.503	1.19E-34	-0.135	2.00E-03	0.047	2.81E-01
**med_ADI_RLL**	-0.332	7.91E-15	-0.095	3.05E-02	-0.381	2.42E-19	0.071	1.06E-01	-0.456	5.72E-28	-0.035	4.26E-01	0.090	4.12E-02
**med_ADI_LUL**	-0.340	1.67E-15	-0.119	6.44E-03	-0.461	1.31E-28	-0.012	7.83E-01	-0.430	8.80E-25	-0.123	5.13E-03	0.057	1.91E-01
**med_ADI_LLL**	-0.297	5.11E-12	-0.116	8.26E-03	-0.413	8.84E-23	0.074	9.02E-02	-0.436	1.88E-25	-0.079	7.16E-02	0.102	2.01E-02
**Dh_sRUL**	-0.044	3.33E-01	0.105	1.91E-02	-0.028	5.31E-01	0.162	3.03E-04	-0.038	4.03E-01	0.063	1.62E-01	0.246	2.85E-08
**Dh_sRML**	-0.159	4.31E-04	0.109	1.63E-02	-0.097	3.15E-02	0.148	1.02E-03	-0.184	4.43E-05	0.106	1.86E-02	0.234	1.63E-07
**Dh_sRLL**	-0.195	1.15E-05	0.104	2.01E-02	-0.079	7.67E-02	0.142	1.52E-03	-0.154	5.62E-04	0.079	7.79E-02	0.145	1.15E-03
**Dh_sLUL**	-0.111	1.34E-02	0.082	6.76E-02	-0.053	2.40E-01	0.062	1.66E-01	-0.115	1.04E-02	0.083	6.31E-02	0.272	7.16E-10
**Dh_sLLL**	-0.118	8.35E-03	0.082	6.95E-02	-0.110	1.40E-02	0.150	7.70E-04	-0.122	6.63E-03	0.048	2.81E-01	0.248	2.17E-08
**WT_sRUL**	0.153	6.65E-04	0.219	9.29E-07	-0.035	4.33E-01	0.212	1.95E-06	-0.064	1.54E-01	0.115	1.03E-02	0.318	4.28E-13
**WT_sRML**	-0.039	3.85E-01	0.209	3.11E-06	-0.056	2.14E-01	0.185	3.94E-05	-0.086	5.66E-02	0.130	4.08E-03	0.198	1.10E-05
**WT_sRLL**	-0.013	7.69E-01	0.196	1.07E-05	-0.085	5.74E-02	0.205	3.96E-06	-0.167	1.91E-04	0.104	2.08E-02	0.240	5.98E-08
**WT_sLUL**	0.021	6.43E-01	0.190	1.91E-05	-0.068	1.28E-01	0.130	3.55E-03	-0.095	3.41E-02	0.118	8.17E-03	0.314	6.84E-13
**WT_sLLL**	0.079	7.68E-02	0.212	1.93E-06	-0.098	2.88E-02	0.205	3.97E-06	-0.134	2.81E-03	0.100	2.52E-02	0.303	4.99E-12
**WA_sRUL**	0.056	2.14E-01	0.163	2.85E-04	0.007	8.72E-01	0.211	2.19E-06	0.021	6.41E-01	0.062	1.72E-01	0.248	2.38E-08
**WA_sRML**	-0.102	2.48E-02	0.182	5.14E-05	-0.087	5.36E-02	0.185	3.89E-05	-0.126	5.33E-03	0.132	3.56E-03	0.232	2.32E-07
**WA_sRLL**	-0.116	9.55E-03	0.133	3.07E-03	-0.080	7.32E-02	0.154	5.59E-04	-0.138	2.07E-03	0.065	1.49E-01	0.147	1.03E-03
**WA_sLUL**	-0.025	5.75E-01	0.164	2.48E-04	-0.056	2.10E-01	0.126	4.93E-03	-0.083	6.52E-02	0.100	2.62E-02	0.301	6.42E-12
**WA_sLLL**	-0.009	8.34E-01	0.168	1.72E-04	-0.089	4.64E-02	0.224	4.48E-07	-0.115	1.03E-02	0.077	8.70E-02	0.302	6.29E-12
**Cr_sRUL**	-0.100	2.58E-02	-0.072	1.11E-01	-0.105	1.98E-02	-0.165	2.39E-04	-0.190	2.12E-05	0.101	2.46E-02	-0.009	8.36E-01
**Cr_sRML**	-0.059	1.90E-01	-0.021	6.36E-01	-0.051	2.60E-01	-0.028	5.43E-01	-0.129	4.25E-03	0.053	2.39E-01	-0.018	6.87E-01
**Cr_sRLL**	-0.076	9.09E-02	-0.061	1.76E-01	-0.035	4.42E-01	-0.018	6.96E-01	-0.089	4.65E-02	0.068	1.28E-01	0.066	1.41E-01
**Cr_sLUL**	-0.127	4.64E-03	-0.048	2.90E-01	-0.018	6.96E-01	-0.094	3.63E-02	-0.186	2.93E-05	0.141	1.58E-03	0.078	8.29E-02
**Cr_sLLL**	-0.050	2.64E-01	-0.093	3.86E-02	-0.138	2.07E-03	-0.156	4.83E-04	-0.146	1.14E-03	-0.014	7.50E-01	-0.033	4.62E-01

Table E 11. Descriptions of the variables listed in **Table E 10.**

Name	Description
ADVAIR	RMU04A8 Inhaled steroids used in last 3 months: Advair (bluticasone/salmeterol)
AEROBID	RMU04A4 Inhaled steroids used in last 3 months: AeroBid (blunisolide)
AGE_DERV_01	Age
ALBUTEROL	RMU05A1 Inhaled bronchodilators used in last 3 months: Albuterol (Proventil, Ventolin, ProAir)
ALBUTEROL_NEB	RMU06A4 nebulized bronchodilators used in last 3 months: albuterol (Proventil, Ventolin, ProAir)
ALBUTEROLIPRATROPIUMBROMIDE	RMU06A3 nebulized bronchodilators used in last 3 months: albuterol and ipratropium bromide (DuoNeb)
APNEA_DIAGNOSED01	Sleep apnea diagnosed by a health professional reported at baseline
ARFORMOTEROL	RMU06A2 nebulized bronchodilators used in last 3 months: arformoterol (Brovana)
AZMACORT	RMU04A1 Inhaled steroids used in last 3 months: Azmacort (triamcinolone)
BMH08B	BMH08B Coronary artery disease
BMH08D	BMH08D Heart attack
BMH08H	BMH08H Congestive heart failure
BMH13A	BMH13A Diabetes
BMI_CM01	BMI (from metric units) (ANT04) at baseline
BUDESONIDEFORMOTEROL	RMU05A14 Inhaled bronchodilators used in last 3 months: budesonide/formoterol (Symbicort)

CB_DIAGNOSED01	Chronic bronchitis diagnosed by a health professional reported at baseline (Yes=1, No=0, Unknowns treated as missing)
CB_VISIT1	Chronic Bronchitis identified at baseline
CBC_BASOPHIL_PCT01	CBC basophil PCT at baseline
CBC_EOSINOPHIL_PCT01	CBC eosinophil PCT at baseline
CBC_LYMPHOCYTE_PCT01	CBC lymphocyte PCT at baseline
CBC_MONOCYTE_PCT01	CBC monocyte PCT at baseline
CBC_NEUTROPHIL_PCT01	CBC Neutrophil PCT at baseline
CBC07_DERV	CBC07 Red blood cell distribution width
CBC08_DERV	CBC08 Total white blood cell count
COPD_DIAGNOSED01	COPD diagnosed by a health professional reported at baseline
COPDSCORE0101	Baseline COPD assessment score
EMPHYSEMA_DIAGNOSED01	Emphysema diagnosed by a health professional reported at baseline
ETHNICITY	Ethnicity (1=Hispanic,0=Non-hispanic) (DEM05 or DEM05a)
FLOVENT	RMU04A5 Inhaled steroids used in last 3 months: Flovent (fluticasone)
FLUTICASONESALMETEROL	RMU05A13 Inhaled bronchodilators used in last 3 months: fluticasone/salmeterol (Advair Diskus)
FORMOTEROL	RMU05A5 Inhaled bronchodilators used in last 3 months: formoterol (Foradil)
FORMOTEROL_NEB	RMU06A1 nebulized bronchodilators used in last 3 months: formoterol (Perforomist)

GENDER	Gender (1=Male,2=Female) (IEC04)
IPRATBROMIALBUTSULFA	RMU05A3 Inhaled bronchodilators used in last 3 months: ipratropium bromide/albuterol sulfate (Combivent)
IPRATROPIUMBROMIDE	RMU05A2 Inhaled bronchodilators used in last 3 months: ipratropium bromide (Atrovent)
IPRATROPIUMBROMIDE_NEB	RMU06A5 nebulized bronchodilators used in last 3 months: ipratropium bromide (Atrovent)
LEVALBUTEROL	RMU05A10 Inhaled bronchodilators used in last 3 months: levalbuterol (Tomalate)
METAPROTERENOL	RMU05A9 Inhaled bronchodilators used in last 3 months: Metaproterenol (Alupent, Metaprel)
OTHERSTEROIDS	RMU04A10 Inhaled steroids used in last 3 months: Other
PEX_SEVERE	total count of exacerbations requiring ED visit or hospitalization since entering the study
PEX_TOT	total count of exacerbations since entering the study
PEX_TOT0101	Total Exacerbations for baseline
PIRBUTEROL	RMU05A8 Inhaled bronchodilators used in last 3 months: Pirbuterol (Maxair)
PULMICORT	RMU04A6 Inhaled steroids used in last 3 months: Pulmicort (budesonide)
PULMONARY_VASCULAR_CONDITION01	History of Pulmonary/vascular condition at baseline
QVAR	RMU04A7 Inhaled steroids used in last 3 months: Qvar (beclomethasone)
RDS08	RDS08 Wheezing/ whistling in chest
RDS12	RDS12 Shortness of breath during sleep
RDS16	RDS16 Asthma

RDS25A3	RDS25A3 Father had COPD
RDS25B3	RDS25B3 Mother had COPD
RMU09A1	RMU09A1 beta blocker used in last 3 months: Atenolol (tenormin, tenoretic)
RMU09A10	RMU09A10 beta blocker used in last 3 months: Other
RMU09A2	RMU09A2 beta blocker used in last 3 months: Metoprolol (lopresor, toprol)
RMU09A3	RMU09A3 beta blocker used in last 3 months: Carvedilol (coreg)
RMU09A5	RMU09A5 beta blocker used in last 3 months: Propranalol (Inderal, Inderide)
RMU09A6	RMU09A6 beta blocker used in last 3 months: Sotalol (Betapace, Sorine)
RMU09A7	RMU09A7 beta blocker used in last 3 months: Timolol (Blocadren, timolide)
RMU09A8	RMU09A8 beta blocker used in last 3 months: bisoprolol (zebeta, ziac)
RMU09A9	RMU09A9 beta blocker used in last 3 months: pindolol (visken)
RMU11	RMU11 Aspirin usage
SALMETEROL	RMU05A7 Inhaled bronchodilators used in last 3 months: Salmeterol (Serevent Diskus)
SIX_MINUTE_WALK_DISTANCE01	Six Minute Walk Distance (m) (from SMW08a,b,c at BASELINE)
SMOKING_PACK_YEARS01	Smoking pack-years at baseline
SMW_DSAT01	Oxygen desaturation with the six minute walk
SYMBICORT	RMU04A9 Inhaled steroids used in last 3 months: Symbicort

TIOTROPIUM	RMU05A6 Inhaled bronchodilators used in last 3 months: tiotropium (Spiriva)
V1_MMP3	Matrix metalloproteinase 3 (stromelysin 1, progelatinase) (MMP3)
WHEEZINGAGE	RDS08A Age of first wheezing/whistling in chest
Dh	Hydraulic luminal diameter
Cr	Airway luminal circularity
WT	Airway wall thickness
WA	Airway wall area
sLLL	Subgrouped left lower lobe with branches of LB6, and LB8 to LB10
sLUL	Subgrouped left upper lobe with branches of LB1 to LB5
sRLL	Subgrouped right lower lobe with branches of RB6 to RB10
sRML	Subgrouped right middle lobe with branches of RB4 to RB5
sRUL	Subgrouped right upper lobe with branches of RB1 to RB3
LLL	Left lower lobe
LUL	Left upper lobe
RLL	Right lower lobe
RML	Right middle lobe
RUL	Right upper lobe

Emphysratio	Emphysema percentage
fSADratio	Functional small airway disease percentage
airtrapratio	Air-trapping percentage
med_J	Median Jacobian value
med_TLC_tissue	Median tissue value
med_ADI	Median Anisotropic Deformation Index value

Table E 12. Indexes of Effect size used in this study and their values for small, medium, and large effects by Cohen's criteria.

	**Small**	**Medium**	**Large**
**Cohen's d**	0.20	0.50	0.80
**Hedge's g**	0.20	0.50	0.80
**Correlation (r)**	0.10	0.30	0.50

Supplementary Reference

1. Costello, A. B. & Osborne, J. W. Best practices in exploratory factor analysis: Four recommendations for getting the most from your analysis. *Pract. Assessment, Res. Eval.* **10**, (2005).

2. Dunlap, J. W. & Thurstone, L. L. The Vectors of the Mind. *Am. J. Psychol.* **49**, 329 (1937).

3. Craig J. Galbán, Meilan K. Han, Jennifer L. Boes, Komal A. Chughtai, Charles R. Meyer, Timothy D. Johnson, Stefanie Galbán, Alnawaz Rehemtulla, Ella A. Kazerooni, Fernando J. Martinez, B. D. R. CT-based Biomarker Provides Unique Signature for Diagnosis of. *Physiol. Behav.* **176**, 139–148 (2017).

4. Choi, S., Hoffman, E. A., Wenzel, S. E., Castro, M. & Lin, C. L. Improved CT-based estimate of pulmonary gas trapping accounting for scanner and lung-volume variations in a multicenter asthmatic study. *J. Appl. Physiol.* **117**, 593–603 (2014).

5. Haghighi, B. *et al.* Imaging-based clusters in current smokers of the COPD cohort associate with clinical characteristics: The SubPopulations and Intermediate Outcome Measures in COPD Study (SPIROMICS) 11 Medical and Health Sciences 1102 Cardiorespiratory Medicine and Haemato. *Respir. Res.* **19**, (2018).

6. Yin, Y., Hoffman, E. A. & Lin, C. L. Mass preserving nonrigid registration of CT lung images using cubic B-spline. *Med. Phys.* **36**, 4213–4222 (2009).

7. Haghighi, B., D. Ellingwood, N., Yin, Y., Hoffman, E. A. & Lin, C. L. A GPU-based symmetric non-rigid image registration method in human lung. *Med. Biol. Eng. Comput.* **56**, 355–371 (2018).

8. Lynch, D. A. *et al.* CT-definable subtypes of chronic obstructive pulmonary disease: A statement of the fleischner society1. *Radiology* **277**, 192–205 (2015).

9. Smith, B. M. *et al.* Human airway branch variation and chronic obstructive pulmonary disease. *Proc. Natl. Acad. Sci. U. S. A.* **115**, E974–E981 (2018).
